# Striatal endocannabinoids drive one-shot learning

**DOI:** 10.1038/s41593-026-02392-z

**Published:** 2026-08-07

**Authors:** Charlotte Piette, Arnaud Hubert, Sylvie Perez, Jérémy Peixoto, Nicolas Gervasi, Hugues Berry, Jonathan Touboul, Laurent Venance

**Affiliations:** 1 Center for Interdisciplinary Research in Biology, Collège de France, CNRS, INSERM, Université PSL, Paris, France; 2 AIstroSight, Inria, Hospices Civils de Lyon, Université Claude Bernard Lyon 1, Villeurbanne, France; 3 Department of Mathematics and Volen National Center for Complex Systems, Brandeis University, Waltham, MA USA

**Keywords:** Long-term memory, Spike-timing-dependent plasticity, Neural decoding

## Abstract

One-shot learning—the ability to form memories after a single, brief salient event—is essential for behavioral flexibility in a dynamic world. However, how one-shot learning unfolds in the brain and whether it relies on distinct plasticity mechanisms remains unknown. Here we show that a nonclassical plasticity mechanism requiring only a few stimulations—endocannabinoid-mediated long-term potentiation (eCB-LTP)—underlies one-shot learning in the striatum. To do so, we developed a one-shot behavioral paradigm—the sticky tape avoidance test—in which mice learn to avoid a piece of sticky tape after a spontaneous single and brief contact. Brief, but not prolonged, contacts drive striatal potentiation in vivo and engage coordinated cortical–striatal activity patterns consistent with eCB-LTP induction, as corroborated by ex vivo electrophysiology and computational modeling. Finally, genetic and pharmacological disruptions of eCB-LTP impaired one-shot learning. These results highlight the essential role of nonclassical plasticity mechanisms in supporting memory formation after a single experience.

## Abstract (web summary)

Endocannabinoid-dependent corticostriatal plasticity underlies avoidance learning in mice after a single experience. Disrupting this process impairs learning, revealing a nonclassical synaptic basis supporting one-shot learning.

## Main

The ability to acquire a long-term memory from a single and brief experience is crucial for behavioral adaptation and is observed in many contexts, supporting spatial learning, recognition memory, complex episodic-like associations, instrumental learning in response to threat or appetitive stimuli, or new word acquisition^1–3^. ‘One-shot learning,’ refers to such learning experiences that involve both a unique and brief exposure to a stimulus. As such, one-shot learning is expected to involve a small number of action potentials or bursts, thereby constraining the neural mechanisms from which it arises^4–9^. Challenges in elucidating its neural underpinnings stem experimentally from the scarcity of rodent behavioral assays of naturalistic one-shot learning, outside of spatial learning and fear/aversion-based tasks and conceptually from the scarcity of plasticity rules compatible with the sparse firing activity or few bursts typical of one-shot experience^10–16^. A notable exception is the hippocampal behavioral timescale synaptic plasticity (BTSP), which has been related to the formation and remapping of place fields during spatial navigation^3,15–18^.

At the corticostriatal synapses in the dorsolateral striatum (DLS), a handful of spikes evoke an eCB-LTP—a form of spike-timing dependent plasticity (STDP)—whereas repetitive stimulation induces an N-methyl-D-aspartate (NMDA) receptor-mediated LTP (NMDAR-LTP)^14,19–22^. We hypothesize that these two distinct molecular pathways could underlie learning in the DLS after a brief one-shot versus longer or repeated experiences, respectively. In vivo, DLS is required in late learning stages^23–25^, which is supported by NMDAR-LTP^26,27^. Long-lasting modulation of striatal neuronal activity has also been reported early in learning^26,28–31^, or following rapid adaptation to abrupt changes in task contingencies^32,33^. Although these behavioral paradigms are not one-shot learning tasks per se, they indicate that striatal projecting neurons (SPNs) can form new associations rapidly from cortical inputs, and adapt motor programs^34,35^. However, whether one-shot learning induces plasticity and learning in corticostriatal circuits has never been investigated directly.

Here we test whether eCB-LTP in the DLS supports one-shot learning. We introduce a new behavioral assay—the sticky tape avoidance (STA) test—in mice and show that a single brief contact induces avoidance upon re-exposure and selectively triggers corticostriatal potentiation in vivo. A data-driven synaptic model indicates that cortical and striatal activity patterns during this brief one-shot learning are sufficient to induce eCB-LTP, consistent with ex vivo occlusion results. Disrupting the eCB-LTP pathway—using conditional knockouts or acute cannabinoid type-1 receptor (CB1R) blockade in the striatum—abolishes one-shot learning. These findings identify eCB-LTP as a mechanistic substrate for rapid, single-trial learning in the DLS.

## Results

### Sticky tape elicits one-shot learning and induces robust memory

**Fig. 1:**
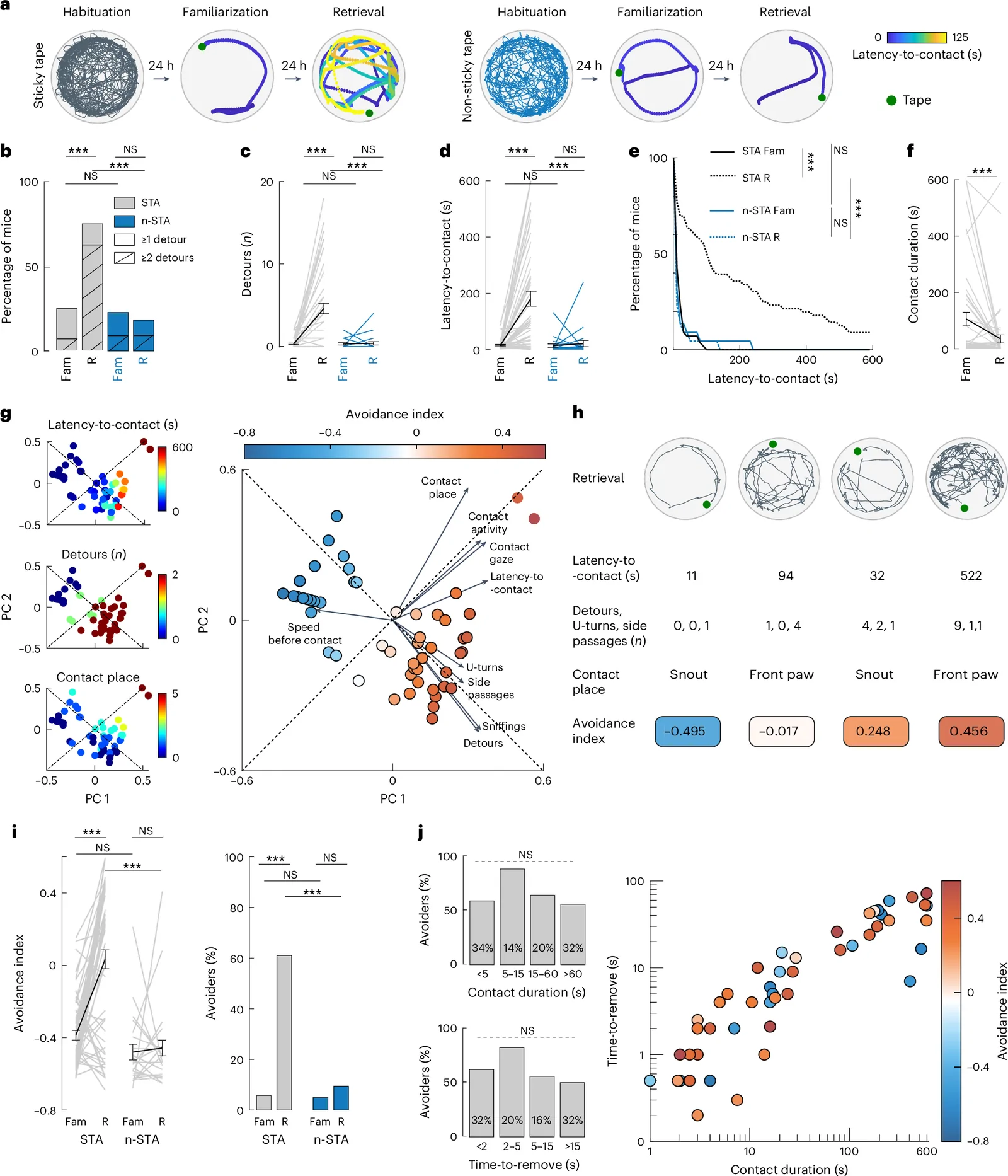
Induction of one-shot learning with the STA test. **a**, Individual mouse trajectory in STA and n-STA tests. For familiarization and retrieval, only the trajectory before the first contact is displayed, and time until contact is indicated in color (the mouse is left 10 min in the arena in all three sessions). **b**–**f**, Mice subjected to STA (*n* = 56) showed increased numbers of detours (**b**,**c**) and latency-to-contact (**d**,**e**) with decreased contact duration (**f**) between familiarization (Fam) and retrieval (R), whereas n-STA mice (*n* = 22) show no changes. Two-sided McNemar test (n-STA Fam/R for ≥1 detour: *P* = 0.705, for ≥2 detours *P* < 0.001; STA: *P* < 0.001 (**b**)), repeated-measures ANOVA with two-sided *t*-tests with Tukey correction (n-STA Fam/R: *p* = 1; STA: *p* < 0.001 (**c**); n-STA Fam/R: *P* = 0.997; STA: *P* < 0.001 (**d**)), log-rank test (n-STA Fam/R: *P* = 0.79; STA: *P* < 0.001 (**e**)) and paired two-sample two-sided Student *t*-test (*P* = 0.002 (**f**)). **g**, Individual mice scoring quantified from a PCA-based avoidance index, computed from a linear combination of the first two PCs, overlaid with parameter values (left) or avoidance index calculated at retrieval (right). Vectors quantify the contribution of each parameter to the first two PCs. **h**, Illustrative trajectories at retrieval of four mice with distinct avoidance indices associated with the detailed scores of critical parameters. **i**, Increase in avoidance index between familiarization and retrieval, and in the percentage of avoiders (positive avoidance index > 0). Repeated-measures ANOVA with post hoc two-sided *t*-tests with Tukey correction (n-STA Fam/R: *P* = 0.995; STA: *P* < 0.001) and two-sided McNemar test (n-STA Fam/R: *P* = 1; STA: *P* < 0.001). **j**, Brief contact duration and time-to-remove (<5 s and <2 s, respectively) at familiarization are sufficient for one-shot learning, and show avoidance indices at retrieval similar to longer contacts. Left: avoiders for different time bins of contact duration and time-to-remove; Percentage: proportion of mice belonging to each time bin. Right: avoidance index at retrieval as a function of contact duration and time-to-remove of the familiarization session. Two-sided chi-squared test. All data: mean ± s.e.m. *P < 0.05; **P < 0.01; ***P < 0.001, NS, nonsignificant (see ‘Statistics’ in Supplementary Information ‘Methods’).

**Extended Data Fig. 1:**
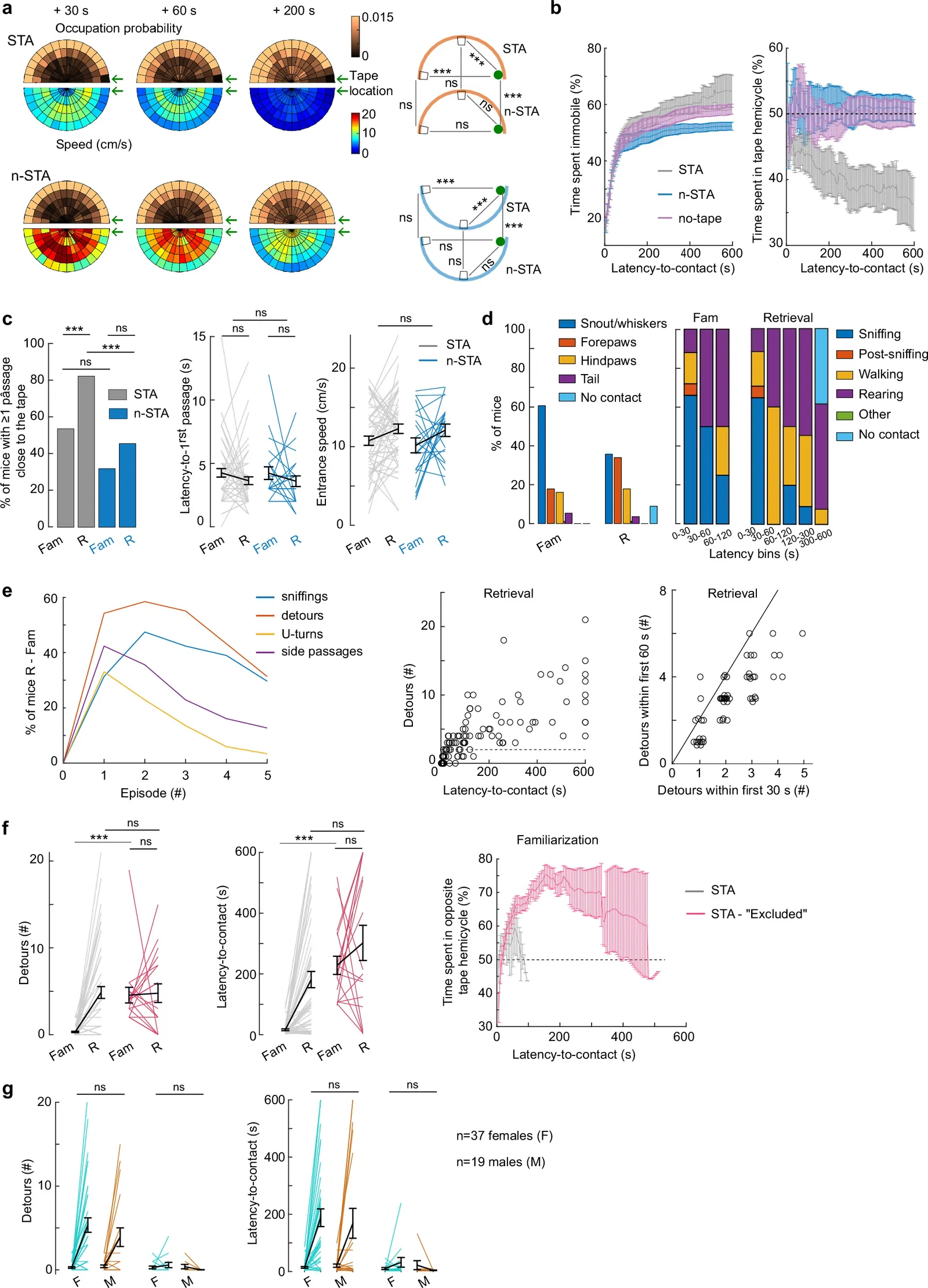
Characterization of the STA and n-STA tests. **a**, Average mouse trajectory in n-STA and STA during retrieval. 2D cumulative heat-maps show occupation probability and average speed. For STA, only mice with latency-to-contact exceeding each timepoint are included (n = 36, 34, 19 mice for 30, 60 and 200 seconds; n-STA: n = 22). Green arrow: tape position. Trajectories are symmetrized relative to the tape position. At retrieval, the sticky tape modifies occupation and speed close to the tape (p < 0.001), but not in n-STA. Linear Mixed Model across zones (close to the tape location in yellow, 90° and 180° opposite wall locations in green and red, respectively) and groups with post-hoc two-sided t-tests (Tukey correction). Cluster variable corresponded to mouse ID. Levels of statistical significance are similar across timepoints. Occupation probability: Fixed Effects of {Group: *F* = 8.822; *p* = 0.003; Timepoints *F* = 8.20, *p* < 0.001; Zone: *F* = 6.86, *p* = 0.001 and Group*Zone: *F* = 17.1, *p* < 0.001}. Average speed: Fixed Effects of {Group: F = 1.26; *p* = 0.263; Timepoints: *F* = 27.7, *p* < 0.001; Zone: *F* = 30.3, *p* = 0.001 and Cluster*Zone: *F* = 14.8, p < 0.001}. Post-hoc t-tests Cluster*Zone with Tukey corrections: STA Scotch/90°: p < 0.001; nSTA Scotch/90°: p = 1.00 for both occupation probability and speed. **b**, Fraction of time spent immobile (right) or in the hemicycle arena containing the tape up to the latency-to-contact (or randomly centered in no-tape condition) (left) show an avoidance behavior at retrieval in STA mice (n = 56), but not in n-STA (n = 22) or no-tape (n = 10) conditions. Linear Mixed Model with post-hoc two-sided t-tests (Tukey correction). For time spent immobile: fixed effect of groups: *F* = 1.022, *p* = 0.036; timepoints: *F* = 0.125, *p* < 0.001 and groups*timepoints: *F* = 0.773, *p* = 0.996; for time spent in the hemicycle arena containing the tape: fixed effects of groups: *F* = 8.02, *p* < 0.001; timepoints: *F* = 4.09, *p* < 0.001 and groups*timepoints: *F* = 0.675, *p* = 1.000. **c**, Left: Non-repelling nature of the tape shown by percentage of mice making at least one passage near the tape before contact. Contingency tables with Chi-squared test at familiarization: χ^2^ = 3.0, *p* = 0.086; Retrieval: χ^2^ = 10.5, *p* = 0.001. McNemar-Test for paired samples: within n-STA (n = 22 mice) for ≥1 passage: χ^2^ = 1.92 *p* = 0.166; within STA (n = 56): χ^2^ = 13.3 *p* < 0.001. Right: latency-to-1^st^ passage and entrance speed do not differ between n-STA and STA mice. Repeated-Measures ANOVA: between subjects: latency first passage *F* = 0.381; *p* = 0.539 (latency-to-first passage); *F* = 0.198, *p* = 0.657 (entrance speed); within subjects (familiarization *vs*. retrieval): *F* = 0.747, *p* = 0.390 (latency-to-first passage); *F* = 5.87, *p* = 0.018 (entrance speed). **d**, Distribution of body part contacting the tape and pre-contact activity (binned by latency-to-contact) at familiarization and retrieval in STA mice. Log-linear regression model: sticking body part (R^2^ = 0.843), familiarization/retrieval predictor: *p* = 1.000; for activity immediately pre-contact (R^2^ = 1), familiarization /retrieval predictor: *p* = 0.004. **e**, Left: Differences in proportions of mice exhibiting a given number of sniffings, detours, U-turns or side passages between familiarization and retrieval. Maximal group difference occurs at 1-2 episodes, defining the avoidance index threshold. Right: detours *vs*. latency-to-contact at retrieval, with sublinear scaling within 30-60 seconds. **f**, In STA, 24% of mice (n = 12 females, n = 6 males) showed avoidance at familiarization; Left: number of detours and latency-to-contact at familiarization between excluded mice (n = 18) and the rest (n = 56). Repeated-Measures ANOVA: between subjects: F = 8.71, p = 0.004 (detours) and F = 30.9; *p* < 0.001 (latency-to-contact). Right: time in the hemicycle opposite to the tape location. Comparison to a 50-50% occupancy at time +30 and +60 seconds: *t* = -3.1, *p* = 0.0063; *t* = -5.9, *p* < 0.001 for exclude mice; *t* = -0.7, *p* = 0.51 and *t* = -1.2, *p* = 0.32 for the rest of mice. **g**, No sex difference in detours and latency-to-contact: males (STA: n = 19, n-STA: n = 8) and females (STA: n = 36, n-STA: n = 16), One-Way ANOVA: n-STA: *F* = 0.925, *p* = 0.348; *F* = 0.428; *p* = 0.520; STA: *F* = 0.796, *p* = 0.376; *F* = 0.0537, *p* = 0.818. Data: mean ± SEM. *p < 0.05; **p < 0.01; ***p < 0.001.

To characterize one-shot learning in mice, we designed a new behavioral assay, the STA test (Fig. 1 and Extended Data Fig. 1). STA relies on spontaneous and unconstrained behavior involving no reward or penalty (Fig. 1a). After habituation to an empty arena, the mouse undergoes a familiarization session in which it explores the arena while a loose piece of sticky tape is positioned on the floor near the walls, with the sticky face up. The mouse inevitably sticks to the tape and attempts to remove it. This single contact with the tape constitutes the one-shot learning experience. Its duration is determined by the mouse’s ability to efficiently detach the tape. In the retrieval session, occurring at least 24 h later, the mouse is reintroduced to the arena with a new piece of sticky tape positioned at a different location. As a control, a similar assay was performed with a piece of nonsticky tape (n-STA test). Note that, at familiarization, the tape constitutes a new object that most mice explore and contact rapidly, with no or very few (one or two) detours, regardless of whether the tape is sticky or not, indicating that the tape is not inherently repellent (Fig. 1b–e).

Learning in this assay manifests itself through the acquisition of an avoidance behavior in the vicinity of the sticky tape at retrieval, which is expressed as a decrease in velocity and the amount of time spent near the tape (Extended Data Fig. 1a,b). A single exposure to the tape was sufficient to elicit avoidance in most mice: at retrieval, we observe a ninefold increase in the proportion of mice making at least two detours around the tape (*n* = 56, McNemar test; *P* < 0.001; Fig. 1b,c). Likewise, whereas 71% of mice contact the tape within the first 15 s at familiarization, this percentage drops to 29% at retrieval (McNemar test; *P* < 0.001; Fig. 1d,e and Extended Data Fig. 1c). Even though 91% of mice still contact the tape again within the 10-min retrieval session, late contacts generally occur with the paws during rearing or walking, preceded by a delayed gaze towards the tape, whereas at familiarization contacts involve mostly the snout and result from direct sniffing (Extended Data Fig. 1d,e). In addition, at retrieval, mice are more efficient at detaching the sticky tape (Fig. 1f). None of the above quantifications changed between familiarization and retrieval in the n-STA control experiment (*n* = 22 mice) (Fig. 1b–e and Extended Data Fig. 1a–c). Of note, in a subset of mice (24%), the first approach to the tape (possibly due to a brief whisker contact and/or higher sensitivity to the stickiness of the tape) triggered an immediate and persistent avoidance behavior towards the tape during familiarization. We excluded these mice from all analyses as their behavior precludes evaluation of learning (Extended Data Fig. 1f). Male and female mice behaved similarly and were grouped together across the whole study (Extended Data Fig. 1g). Together, these data demonstrate that a single encounter with a sticky tape is sufficient to create a long-term memory.

**Extended Data Fig. 2:**
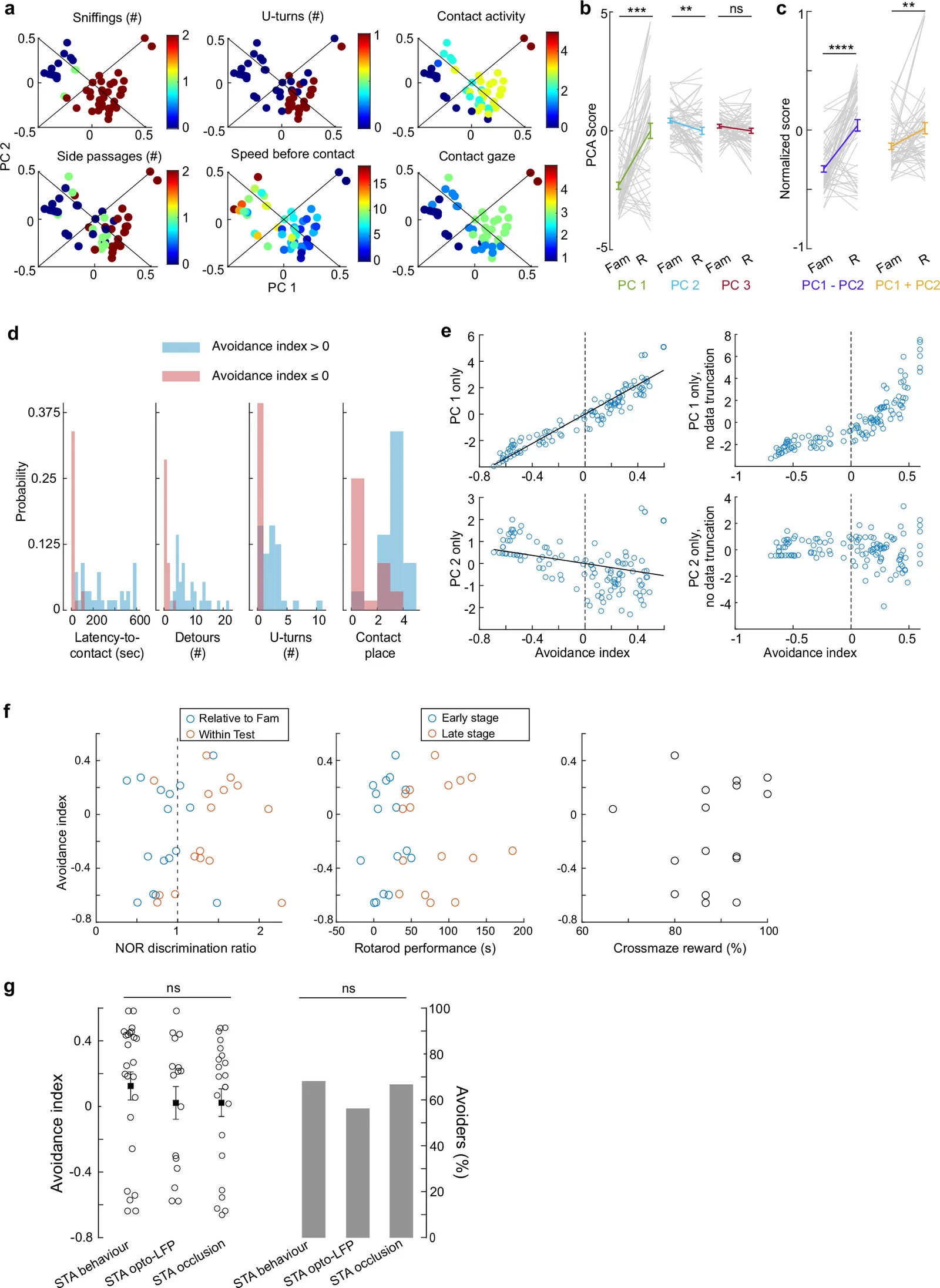
Evaluation of one-shot learning performance and its robustness. **a-c**, One-shot learning avoidance index. **a**, Six additional panels showing behavioral parameter distributions during retrieval in PCA space, as in Fig. 1g. **b**, Individual mouse scores on the first three PCs differ between familiarization and retrieval for PC1 (green, *t* = -6.45, *p* < 0.001) and PC2 (blue, *t* = 3.31, *p* = 0.002), but not PC3 (*t* = 0.088, *p* = 0.93) (two-sided paired two-sample t-tests, n = 56 mice). **c**, Linear combinations {PC1-PC2} (purple) and {PC1 + PC2} (yellow) capture most variance and increase between familiarization and retrieval with distinct steepness (PC1-PC2: *t* = -7.0, *p* < 0.001; PC1 + PC2: *t* = -3.01, *p* = 0.004) (two-sided paired two-sample t-tests, n = 56 mice). Their ratio (0.45) weights the contribution of each linear combination in the avoidance index (*Methods*). **d**, Probability distribution of key behavioral parameters learner mice (avoidance index >0) or non-learners. **e**, Correlation of avoidance index and PC1 (*r* = 0.945, *p* < 0.0001) and PC2 (*r* = -0.32, *p* = 0.0003) for truncated (left) and non-truncated (right) input data. **f**, In the {1 month + extra-tasks} configuration, STA performance does not correlate with NOR, crossmaze and rotarod learning. Left: NOR discrimination ratio: blue circles for time spent on the novel object at Test / average time spent on novel objects at familiarization; orange for time spent on the novel object at Test / average time spent on familiar objects at Test. Middle: Accelerating rotarod performance: the indices subtract average performance of the last 2 trials of Day 1 (early stage), or the last 2 trials of Day 5 (late stage) to the average of the first 2 trials of Day 1. *Right*: Crossmaze performance (% of rewarded trials, final day). Correlations between avoidance index and NOR discrimination ratio relative to familiarization and within respectively (Pearson’s r = 0.08 and 0.22; *p* = 0.768 and 0.424 respectively; two-sided Pearson correlation test); early and late rotarod performance (Pearson’s r = 0.17 and 0.018; *p* = 0.537 and 0.949 respectively; two-sided Pearson correlation test); crossmaze performance (Pearson’s r = 0.11, *p* = 0.679; two-sided Pearson correlation test). **g**, Avoidance index and avoider rate (grey) at retrieval (24 h from familiarization) did not differ across STA behavior only (n = 24 mice), *in vivo* opto-LFP (n = 16) and *ex vivo* occlusion (n = 12) groups; One-way ANOVA for avoidance index: *F* = 0.46, *p* = 0.6354; Two-sided Chi-squared test for avoider rate: χ^2^ = 0.488 *p* = 0.784. All data: mean ± SEM. *p < 0.05; **p < 0.01; ***p < 0.001.

To score individual mouse avoidance precisely at retrieval, we performed an in-depth analysis of the mouse behavior that captures its variation and richness. To this end, we designed a principal component (PC) analysis (PCA)-based avoidance index that incorporates behavioral features both before and during contact with the sticky tape (latency-to-contact, detours, sniffings, U-turns, side passages, speed in the 2 s preceding contact, activity and gaze immediately before contact and body part contacting the tape) (Fig. 1g,h, Extended Data Fig. 2a–c, Supplementary Information ‘Methods’, Supplementary Note and Supplementary Figs. 1–3; Methods). The index was scaled so that negative values of the avoidance index reflect no avoidance behavior whereas positive values correspond to elevated latency-to-contact, at least two detours and distracted contacts (for example, during rearing or walking at high speed), allowing to discriminate avoiders (positive index) from nonlearners (negative index). Consistent with the above observations, the avoidance index clearly distinguished behaviors at familiarization and retrieval in STA (paired *t*-test; *P* < 0.001), with 63% of the mice categorized as avoiders at retrieval (Fig. 1i and Extended Data Fig. 2d). The avoidance index closely matches other behavioral metrics (such as the latency-to-contact or PC1), demonstrating that these observations are robust to the choice of behavioral index (Extended Data Fig. 2d,e).

One-shot learning is characterized both by the uniqueness of a given experience and its brevity. To explore the lower temporal limits of one-shot learning in the STA test, we investigated whether the avoidance index at retrieval correlated with the duration of contact with the sticky tape and the time-to-remove (the cumulative duration of active tape removal) at familiarization. We found that contacts below 5 s induced avoidance in 58% mice, similar to what was observed with more prolonged contact (Fig. 1j). Extended contact with the tape (>1 min) did not increase the proportion of avoiders, suggesting that learning in this assay quickly asymptotes after just a few seconds of contact. Thus, the STA test is particularly well suited to exploring whether distinct learning mechanisms are recruited following a unique brief versus a long experience.

**Extended Data Fig. 3:**
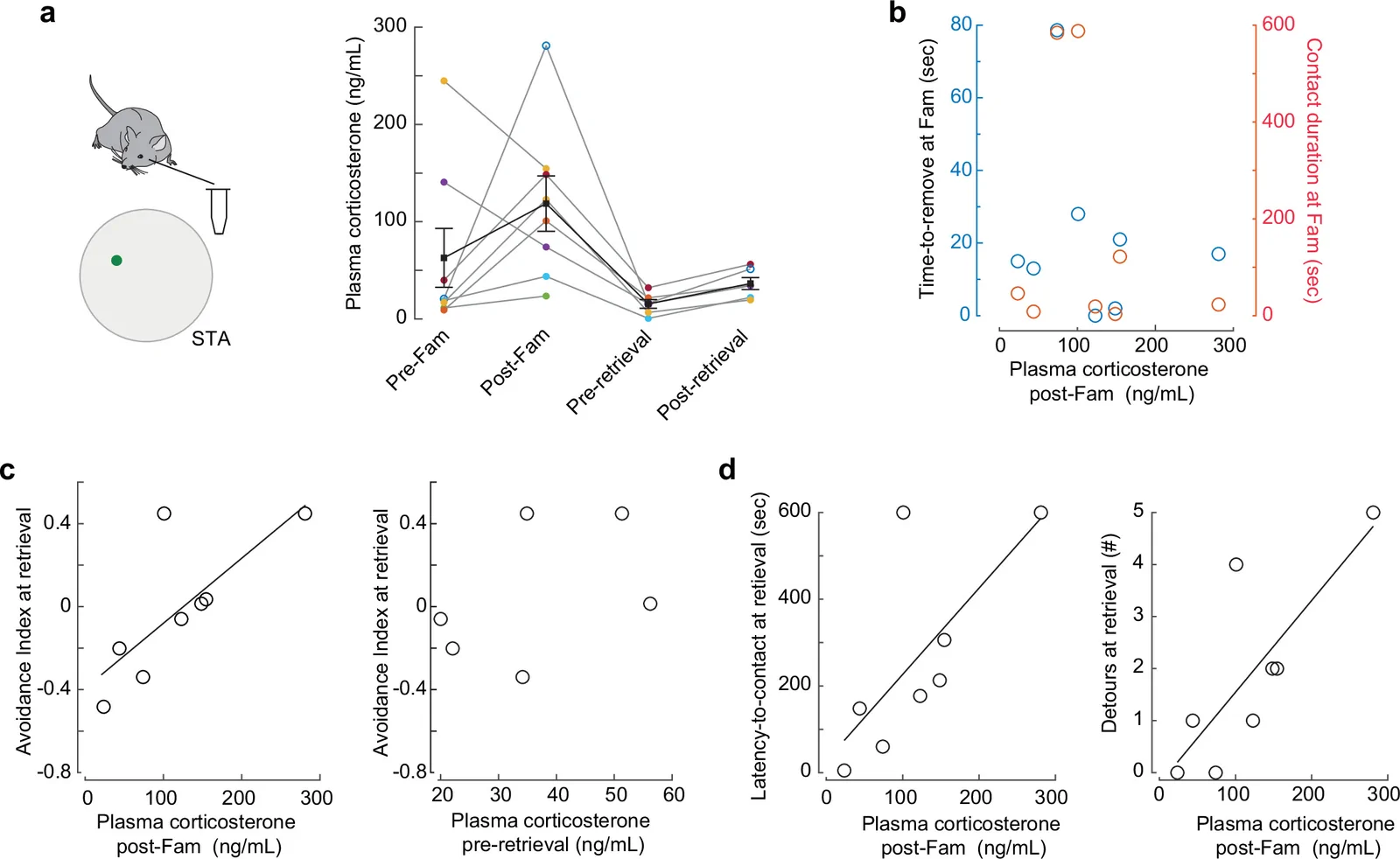
Corticosterone levels during STA correlate with one-shot learning of the STA test. **a**, Plasma concentration of corticosterone at different timepoints before (30 min) and after (20 min) familiarization and retrieval. A tendency towards increased corticosterone levels just after at familiarization can be noted (*t* = -1.41, *p* = 0.2010, paired two-sided t-test, n = 8 mice). **b**, The plasmatic corticosterone level did not depend on the time-to-remove or the contact duration with the sticky tape at familiarization. **c**,**d**, Correlation between the plasmatic levels of corticosterone and the avoidance index (at retrieval) was significant just after familiarization (*r* = 0.75, *p* = 0.032, Pearson correlation two-sided test) but not before retrieval (**c**). Similarly, plasmatic levels of corticosterone after familiarization correlated with the latency-to-contact (*r* = 0.708, *p* = 0.0493, Pearson correlation two-sided test) and the number of detours at retrieval (*r* = 0.786, *p* = 0.0208) (**d**), indicating that one-shot learning depends on the saliency of the one-shot experience. All data: mean ± SEM. *p < 0.05; **p < 0.01; ***p < 0.001.

To test whether STA test induces stress, and whether it relates to the learning quality, we measured the corticosterone plasma concentrations (*n* = 8 mice) (Extended Data Fig. 3). Corticosterone levels did not increase significantly after initial tape exposure or retrieval, suggesting that the sticky tape does not elicit sustained stress and is unlikely to be aversive (Extended Data Fig. 3a,b). However, absolute corticosterone concentrations after, but not before, familiarization were correlated with the avoidance index at retrieval (*r* = 0.750; *P* = 0.032) (Extended Data Fig. 3c,d). These results suggest that successful learning may be influenced by the saliency of, and level of stress induced by, the one-shot experience.

**Fig. 2:**
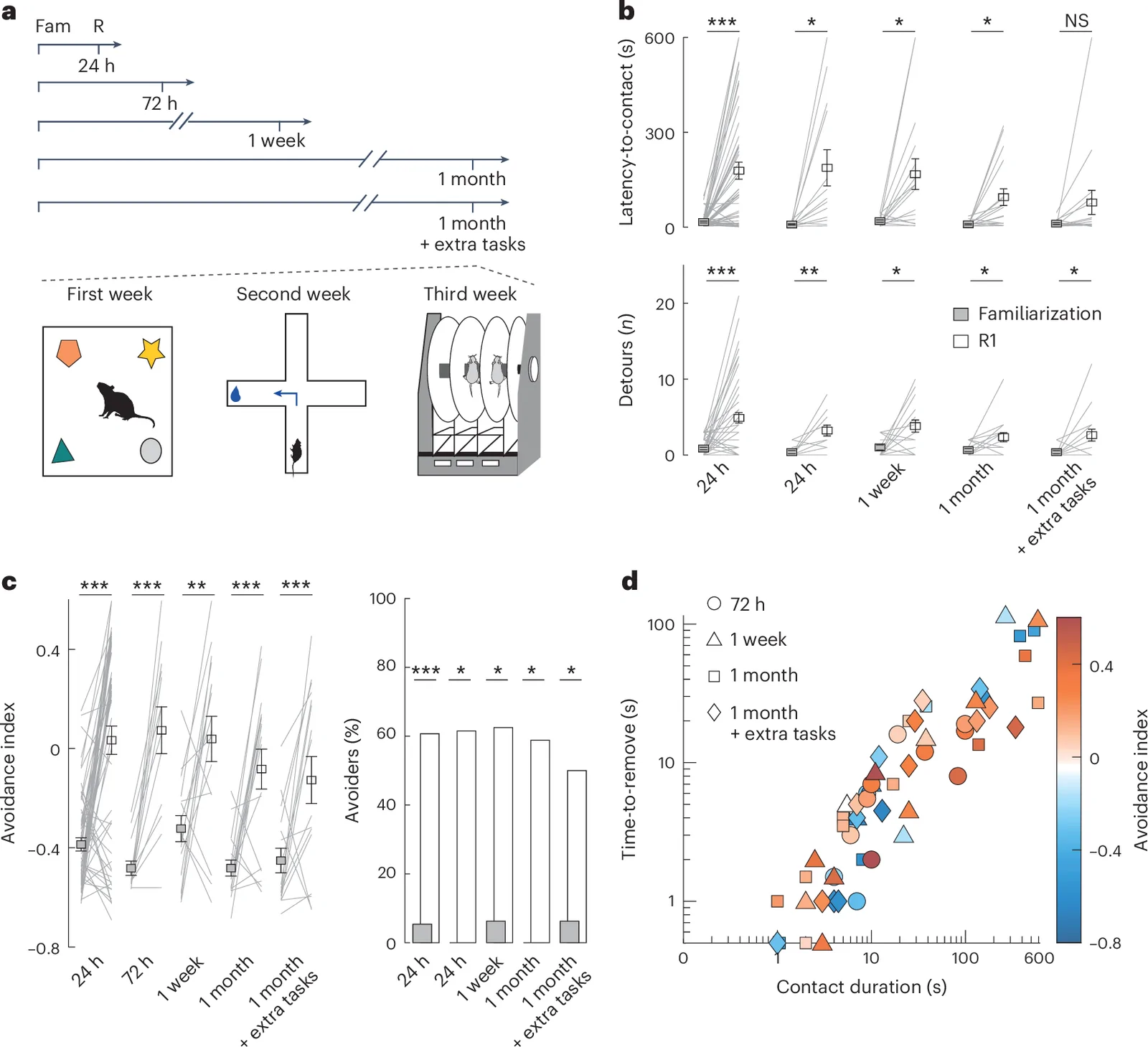
Robustness over time of one-shot learning in the STA test. **a**, Long-lasting effects of a one-shot experience of the STA test, assessed with four increasing time retention intervals (24 h, *n* = 56 mice; 72 h, *n* = 13; 1 week, *n* = 16; 1 month, *n* = 17) between familiarization and retrieval, and over time (1 month) with extra tasks (*n* = 16 mice) (new object recognition, cross-maze and accelerating rotarod). **b**,**c**, Latency-to-contact and number of detours (**b**) and avoidance index and percentage of avoiders (**c**) as a function of the delay since first exposure. Metrics in **b** and **c** show significant differences between familiarization and retrieval for each of the five conditions (paired *t*-tests with Benjamini–Hochberg correction; for avoidance index: 24 h: *P* < 0.001, 72 h: *P* < 0.001, 1 week: *P* = 0.00624, 1 month: *P* = 0.00121 and 1 month+ extra tasks: *P* = 0.00624). **d**, Contact duration does not affect learning performance when retention intervals are introduced between familiarization and retrieval (ANCOVA). Avoidance index at retrieval distribution relative to contact duration and time-to-remove at familiarization. All data: mean ± s.e.m. *P < 0.05; **P < 0.01; ***P < 0.001 (see ‘Statistics’ in Supplementary Information ‘Methods’).

To assess the persistence of the memory created after one-shot learning, three groups of mice were tested with increasing time intervals between familiarization and retrieval. We observed that after 72 h (*n* = 13 mice), 1 week (*n* = 16 mice) or even up to 1 month (*n* = 17 mice), mice still exhibited marked avoidance of the sticky tape, when retrieval features (latency-to-contact, detours, avoidance index and percentage of avoiders) were compared to familiarization, regardless of the contact duration (Fig. 2). An additional cohort of mice (*n* = 16) was subjected to three striatal-dependent learning tasks (accelerating rotarod, cross-maze and new object recognition) between familiarization and the 1-month delayed retrieval. Here again, significant differences in detours, avoidance index and in the percentage of avoiders (but not in latency-to-contact) were observed between familiarization and retrieval, whereas retrieval behavior was similar to that of the 1-month delay interval (Fig. 2). Moreover, STA avoiders and nonlearners performed equally well on the three additional tasks (Extended Data Fig. 2f). These results demonstrate that STA-induced one-shot memory is persistent over time and resists interferences from other long-term memory tasks.

### One-shot learning induces in vivo corticostriatal potentiation

**Fig. 3:**
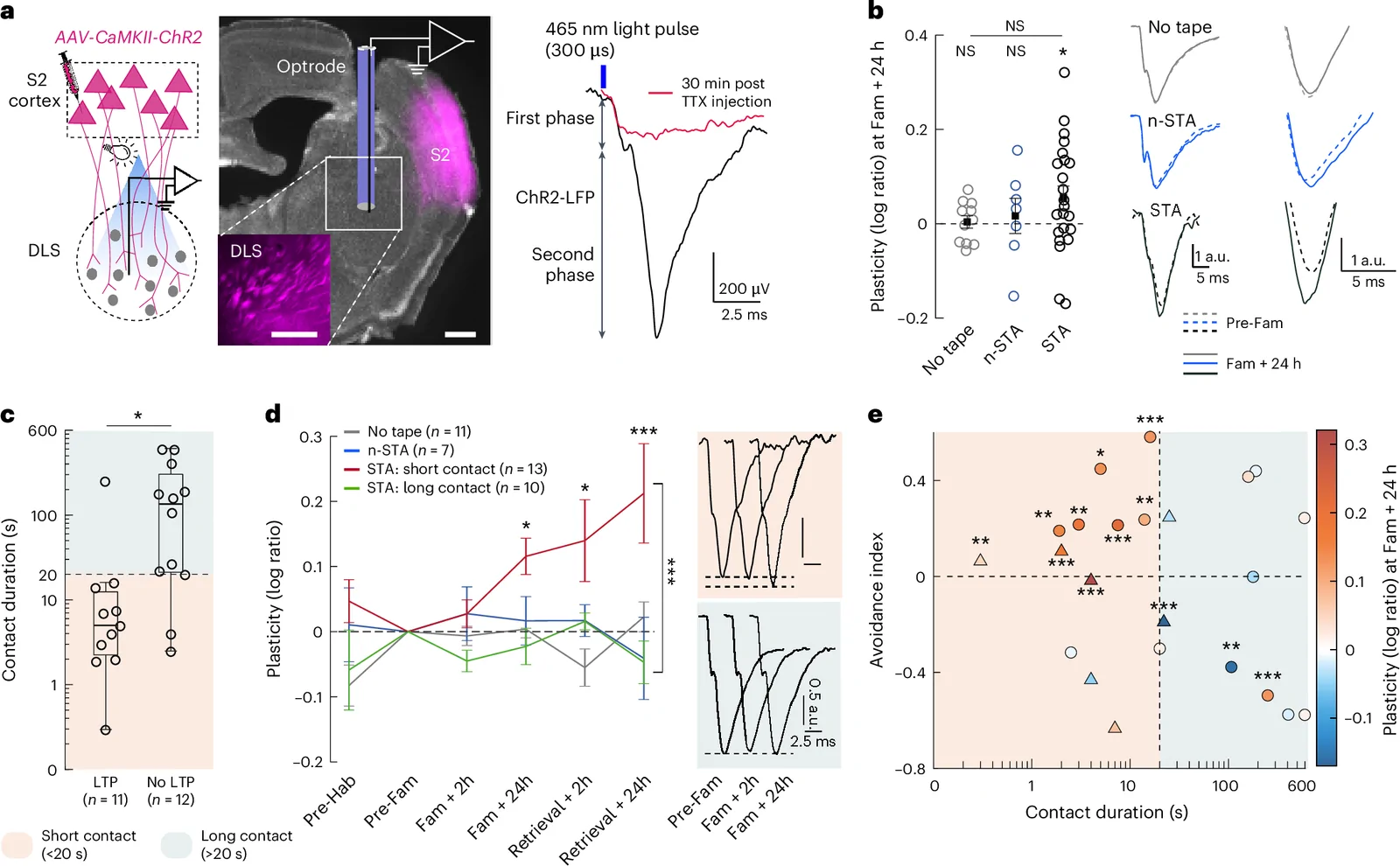
A single and brief exposure to the sticky tape induces in vivo cortical–striatal plasticity, the expression of which correlates with learning. **a**, ChR2-LFP monitoring to assess in vivo corticostriatal plasticity (Methods). Virus expression (magenta) and optrode, zooming on secondary somatosensory cortical terminals in DLS. Scale bars, 0.5 mm. Illustrative ChR2-LFP raw trace from a single light-pulse stimulation. ChR2-LFP displays an early phase (latency-to-peak: 0.77 ± 0.04 ms, *n* = 41), due to light-evoked ChR2 currents and a late phase (latency-to-peak: 2.7 ± 0.1 ms) accounting for synaptic currents. In vivo injection of TTX (1 μM, 1 μl, *n* = 4 mice) prevents expression of the late phase of ChR2-LFP. **b**, Scatterplot of plasticity log ratio 24 h postfamiliarization shows a significant corticostriatal LTP of ChR2-LFP specifically in STA test (*n* = 23 mice; *P* = 0.0327), but not for the no-tape (*n* = 11; *P* = 0.781) and n-STA conditions (*n* = 7; *P* = 0.671). One-way ANOVA and two-sided one-sample Student *t*-test. Representative ChR2-LFP traces at prefamiliarization and 24 h postfamiliarization. a.u., arbitrary unit. **c**, Contact duration with the sticky tape at familiarization discriminates between mice exhibiting ChR2-LFP potentiation or not (dashed line: threshold value of 20 s). Two-sided two-sample Student *t*-test (*P* = 0.0295). Boxplots show the median as the central mark; the box spans from the 25th to the 75th percentile (IQR) and whiskers extend to 1.5× IQR. **d**, Averaged timecourses of plasticity log ratio (normalized by the ChR2-LFP amplitude at prefamiliarization) for STA mice depending on contact duration (threshold ≤20 s), no-tape and n-STA. Representative ChR2-LFP traces at prefamiliarization and familiarization +2 and +24 h for short (*n* = 13) and long (*n* = 10) contacts. Linear mixed model with Tukey correction applied to post hoc two-sided *t*-tests. **e**, Plasticity log ratio at 24 h postfamiliarization (color-coded) as a function of STA avoidance index at retrieval and contact duration at familiarization shows that all mice displayed ChR2-LFP potentiation after short contact leading to high avoidance index. Circles and triangles represent mice after 24 h or 48 h and 1 week delays, respectively, between familiarization and retrieval. Two-sided one-sample Student *t*-test and ANCOVA. All data: mean ± s.e.m. *P < 0.05; **P < 0.01; ***P < 0.001 (see ‘Statistics’ in Supplementary Information ‘Methods’).

**Extended Data Fig. 4:**
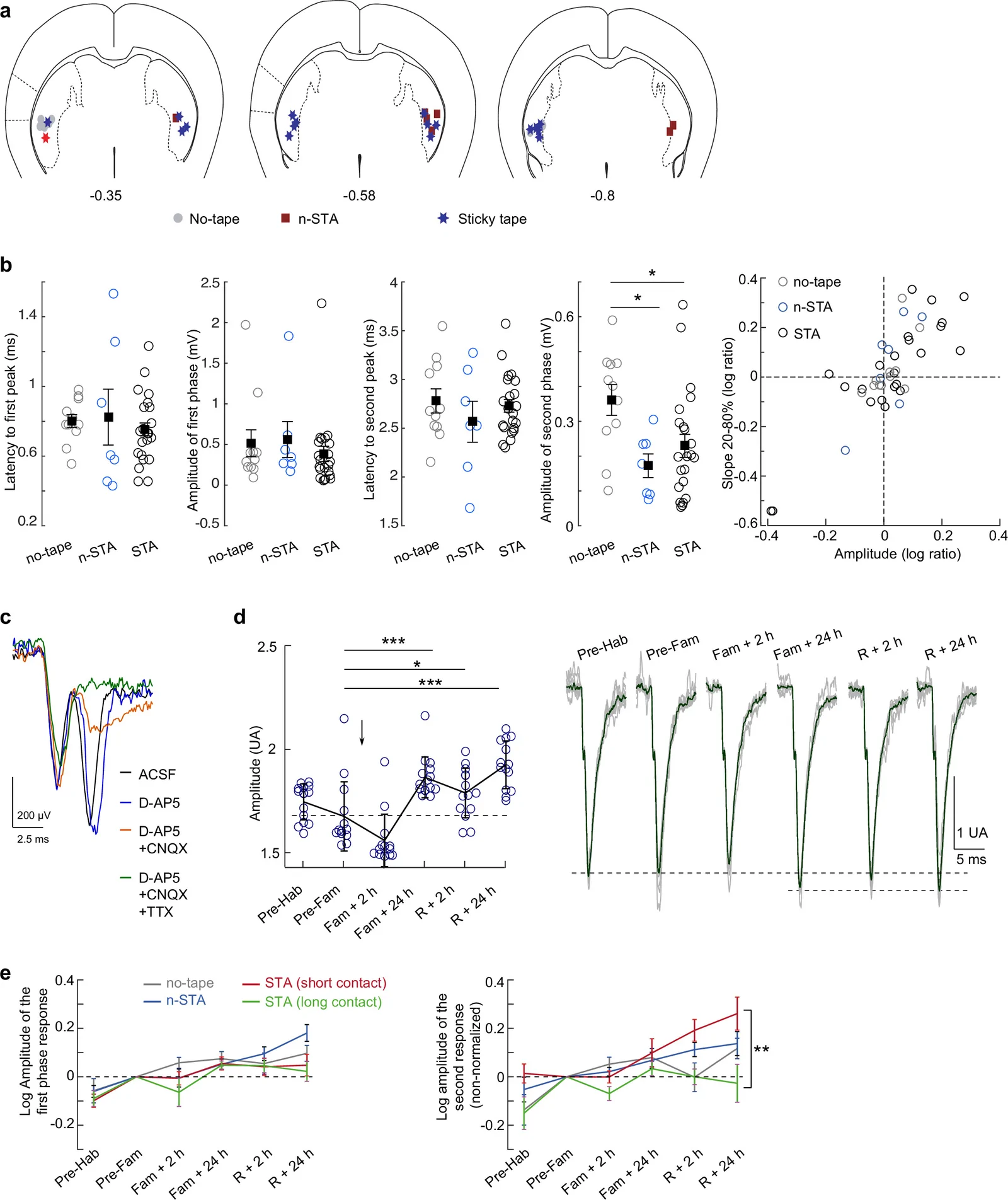
ChR2-LFP characterization. **a**, Recording sites in DLS on three coronal slices (0.3, 0.58 and 0.8 mm posterior to bregma). No-tape, n-STA and STA mice are represented with grey circles, red squares and blue stars, respectively. **b**, *Left*: Latency and amplitudes of early and late ChR2-LFP phases across groups (One-way ANOVA: latency 1^st^: *F* = 0.309, *p* = 0.739; amplitude 1^st^: F = 0.309, *p* = 0.739; latency 2^nd^: *F* = 0.368; *p* = 0.699; amplitude 2^nd^: *F* = 4.689, *p* = 0.015; post-hoc t-tests with Tukey correction: amplitude 2^nd^ no-tape *vs*. n-STA: *p* = 0.0285; no-tape *vs*. STA: *p* = 0.0320; n = 23 STA-mice, n = 7 n-STA mice, n = 11 no-tape mice). *Right*: Plasticity ratios obtained at familiarization +24 h, using the amplitude or slope (20-80%) of the average normalized response, are consistent (Pearson correlation two-sided test, r = 0.909, *p* < 0.001). **c**, Pharmacological blockade *in vitro* shows a strong reduction of the late phase of ChR2-LFP under D-AP5 (50 mM) and CNQX (1 mM) and a complete abolishment under TTX (1 μM). **d**, Example of chronic recordings during STA. Left: amplitude (mean ± SD) of the second phase (measured on the response normalized by the amplitude of the first phase). Each dot represents the amplitude on the average of 6 consecutive stimulations. One-way ANOVA: *F* = 33.9, *p* < 0.001 (post-hoc t-tests with Tukey correction: pre-Fam *vs*. Fam+24 h: *p* < 0.001; pre-Fam *vs*. R + 2 h:; pre-Fam *vs*. R + 24 h: *p* < 0.001). Right: Illustrative traces from the same mouse (grey traces: averages of 6 consecutive stimulations; dark trace: total average). **e**, Average time course of the log ratio of the amplitude of the first phase (left) or second phase (right), non-normalized, of the ChR2-LFP response for all four groups of mice. Linear Mixed Models (two factors: Group, Timepoints; cluster variable corresponds to mouse ID) on log ratio of the amplitude of the first phase (Fixed Effect of Group: F = 1.51, p = 0.227; Time: F = 17.21, *p* < 0.01; Group*Time: F = 1.06, p = 0.398) or second phase (Fixed Effect of Group: F = 5.14, *p* = 0.005; Time; F = 13.01, *p* < 0.01; Group*Time: F = 1.98, *p* = 0.019). Post hoc two-sided tests (Bonferroni correction) on the second phase: STA-short (n = 11 mice) *vs*. STA-long (n = 12) t = 3.84, *p* = 0.003; STA-long *vs*. Ctrl: t = 1.645, *p* = 0.651; STA-short *vs*. Ctrl: t = -2.262, *p* = 0.178. All data (except **d**): mean ± SEM. *p < 0.05; **p < 0.01; ***p < 0.001.

The striatum has been implicated in controlling the choice between approach and avoidance^36,37^, which is modified by experience in the STA assay. To test whether corticostriatal synapses are engaged in vivo after a one-shot experience with the sticky tape, we monitored corticostriatal plasticity in the DLS. To this end, we optostimulated somatosensory cortical terminals expressing channelrhodopsin (ChR2) and recorded the evoked local field potential (ChR2-LFP) in the DLS with a chronically implanted optrode before and after each behavioral session (Fig. 3 and Extended Data Fig. 4a). ChR2-LFP is composed of two main phases, namely an early fast component (a combination of the photoelectric artifact and light-evoked ChR2 axonal currents) and a late and slower component, that is, the synaptic currents^38^ (Fig. 3a and Extended Data Fig. 4b). We confirmed that the late phase of ChR2-LFP largely reflected excitatory synaptic currents, as it was reduced by AMPA and NMDA receptors blockade, and fully abolished after inhibition of voltage-dependent sodium channels (Fig. 3a and Extended Data Fig. 4c). We used the amplitude of the late phase of ChR2-LFP (normalized by early phase amplitude) as a proxy for corticostriatal plasticity in vivo^38^.

**Extended Data Fig. 5:**
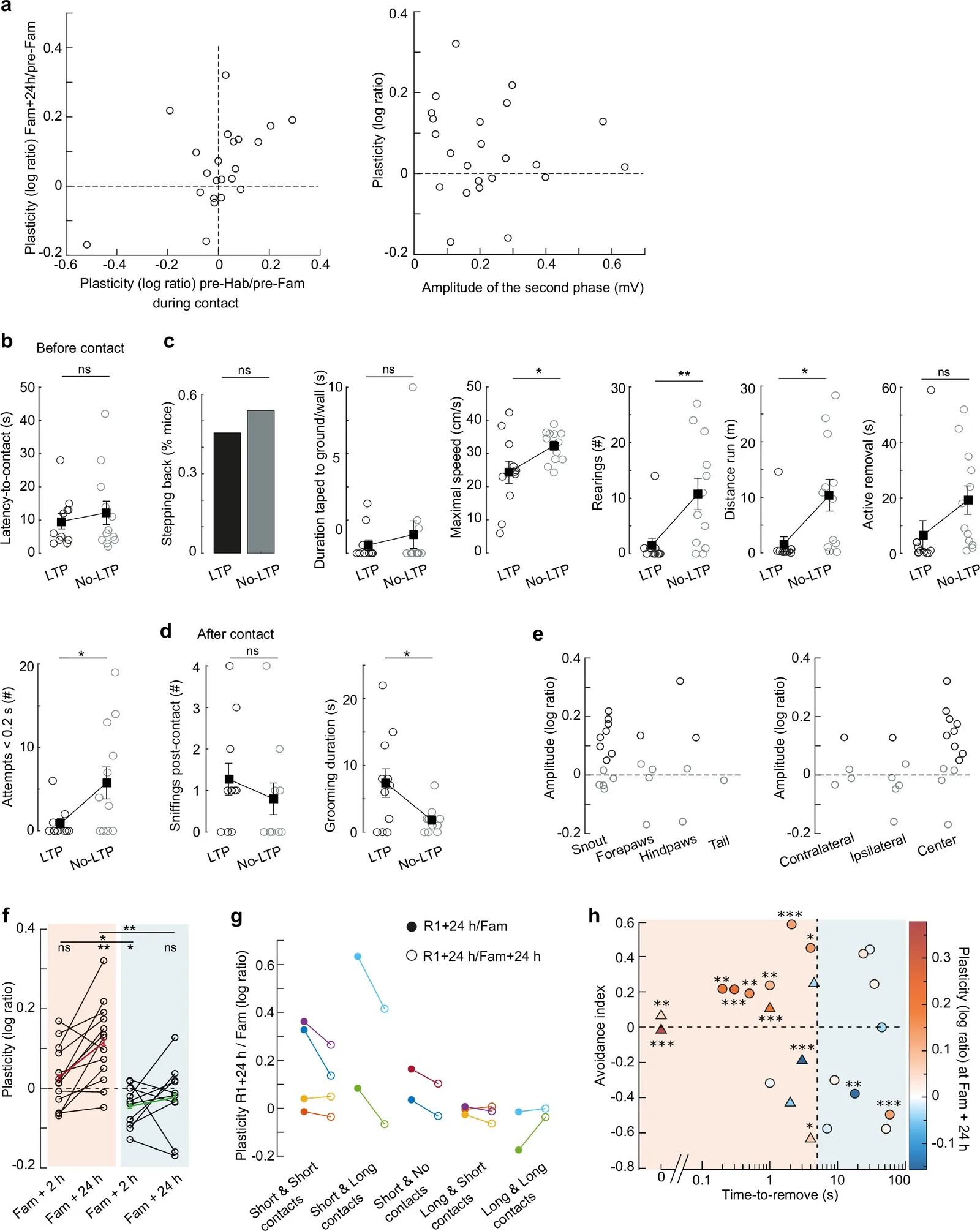
Relation between ChR2-LFP plasticity and behavioral features. **a**, Left: Plasticity ratio at familiarization +24 h shows no correlation with plasticity ratio between Pre-habituation/Pre-familiarization (informing about potentiation expression following habituation, corresponding to a ratio <1) (Pearson’s r = 0.286, *p* = 0.1973). Right: Absence of correlation between plasticity at familiarization +24 h and amplitude of second phase of ChR2-LFP at familiarization (Pearson’s r = -0.1030, *p* = 0.6399). **b-e**, Dependence of ChR2-LFP on behavioral parameters at familiarization (n = 23 mice): (**b**) before, (**c**) during, and (**d**) after contact. Significant differences (two-sample two-sided independent t-tests) were observed during contact for the distance travelled (*p* = 0.013), the maximal speed (*p* = 0.032), rearings (*p* = 0.009), very short ( < 0.2 second) attempts at removing the sticky tape (*p* = 0.029) and the time spent in grooming 1 minute after contact (*p* = 0.027). **e**, Left: Body part stuck to the sticky tape does not influence ChR2-LFP potentiation expression (two-sided Chi-squared test, χ^2^ = 3.46, *p* = 0.327). Right: LTP for all three contact positions relative to recording side (two-sided Chi-squared test, χ^2^ = 5.56, *p* = 0.062). **f**, Plasticity ratio at familiarization +2 h and familiarization + 24 h for short and long contact (threshold ≤20 seconds). One-sample two-sided t-test: mice with short contact at familiarization +2 h: {*t* = 1.36, *p* = 0.20}; short-familiarization +24 h: {*t* = 4.01, *p* = 0.0017}; long-familiarization +2 h: {*t* = -2.73, *p* = 0.023}; long-familiarization +24 h: {*t* = -0.70, *p* = 0.50}. Two-sample two-sided t-tests between short and long contact duration at familiarization +2 h: {*t* = 2.52, *p* = 0.020} and at familiarization +24 h: {*t* = 3.38, *p* = 0.0028}. **g**, Plasticity ratio measured at retrieval +24 h normalized either by familiarization (filled) or familiarization +24 h (empty) depending on the contact duration at familiarization and retrieval. Most of mice showed stable plasticity profiles between familiarization +24 h and retrieval +24 h, and in particular mice (n = 3) with long and then short contact duration at familiarization and retrieval, respectively, did not express ChR2-LFP potentiation at retrieval +24 h. **h**, Plasticity ratio at familiarization +24 h (color-coded) as a function of STA avoidance index and time-to-remove. Circles and triangles: mice with delays between familiarization and retrieval of 24 h and longer (48 h or 1 week), respectively. Statistical significance of each LTP is indicated (one-sample two-sided t-test). ANCOVA on avoidance with two binary control variables {significant LTP and time-to-remove (≤5 seconds)}: no effect of each control variable (*F* = 0.043 and *F* = 1.61 respectively) and combined control variables (*F* = 2.69, *p* = 0.117). All data: mean ± SEM. *p < 0.05; **p < 0.01; ***p < 0.001.

Chronic in vivo recordings of corticostriatal plasticity were performed either in mice placed in an empty arena (no tape, *n* = 11), or subjected to the n-STA (*n* = 7) or STA (*n* = 23) test (Fig. 3b and Extended Data Fig. 4d,e). In both control groups (no tape and n-STA), we did not observe a significant change in the amplitude of the late phase of the ChR2-LFP 24 h postfamiliarization. In contrast, we observed heterogeneous changes in ChR2-LFP amplitude across animals in the STA condition, with a potentiation detected in half of the mice (Fig. 3b and Extended Data Fig. 5a). Despite this variation, a ChR2-LFP potentiation was expressed on average 24 h postfamiliarization (one-sample *t*-test; *P* = 0.023). To examine the origins of this heterogeneity, we correlated in vivo plasticity to behavior at familiarization and retrieval (Extended Data Fig. 5b–e). We found that ChR2-LFP potentiation occurred only in mice with short contact durations (2–20 s, 91% of mice expressing potentiation, *n* = 11), whereas 75% of the mice that did not express potentiation of ChR2-LFP (*n* = 12) contacted the tape for longer than 20 s (independent *t*-test at +24 h, *P* = 0.03; linear mixed model, *P* = 0.003) (Fig. 3c,d and Extended Data Fig. 5b–f). Similarly, those mice that expressed ChR2-LFP potentiation removed the tape actively, with removal times ranging from 0.2 to 15 s (Extended Data Fig. 5c). Once evoked, potentiation of ChR2-LFP persisted for at least 48 h (Fig. 3d) and no potentiation was elicited following the second exposure to the tape (at retrieval +24 h), even if the contact was brief (Extended Data Fig. 5g). Finally, all mice that experienced a short contact and expressed a significant potentiation of ChR2-LFP showed a high avoidance index at retrieval (analysis of covariance (ANCOVA); *P* = 0.041) (Fig. 3e and Extended Data Figs. 2f and 5h). For longer contact durations (>20 s), avoidance of the sticky tape could be observed despite the absence of potentiation of ChR2-LFP in the DLS (Fig. 3c–e). These results demonstrate that a potentiation emerges and persists in corticostriatal circuits following a new single and brief experience as short as ~1 s, and that ChR2-LFP potentiation expression correlates with successful one-shot learning. The absence of potentiation of ChR2-LFP in the DLS after a more prolonged experience suggests that distinct mechanisms or brain circuits are involved.

### In vivo corticostriatal activity during STA is compatible with eCB-LTP expression

**Fig. 4:**
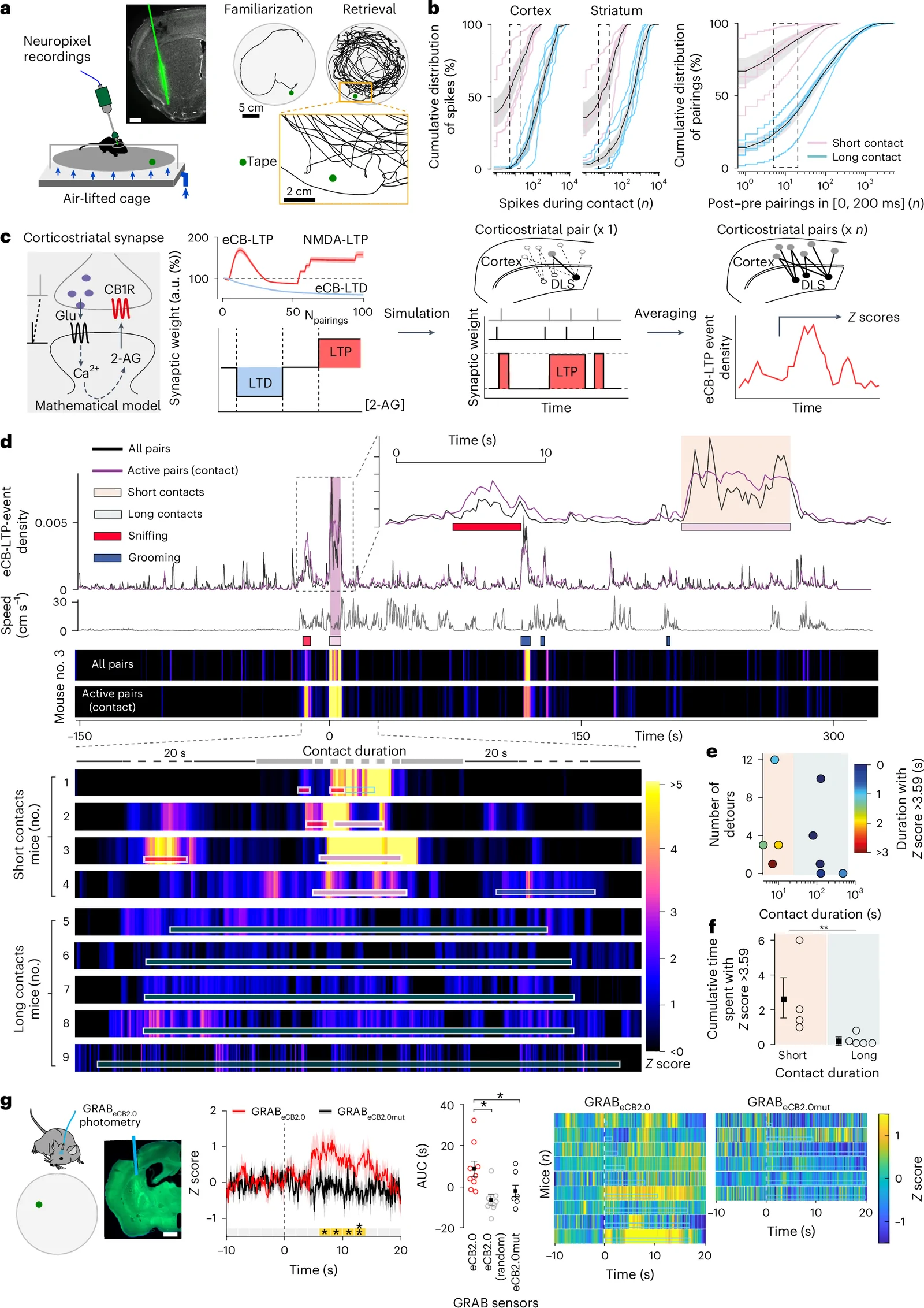
In vivo corticostriatal activity during brief contact engages eCB-LTP. **a**, Acute Neuropixel-1.0 recordings in head-fixed mice in the MHC (*n* = 9 mice). Coronal section showing Neuropixel trajectory targeting primary somatosensory cortex and DLS. Scale bar, 0.5 mm. Illustrative mouse trajectories with inset zoom on detours at retrieval. **b**, Short contacts (*n* = 4 mice) show low activity and sparse post–pre pairings, consistent with in vitro eCB-LTP, unlike long contacts (*n* = 5). Left: cumulative percentage of neurons displaying at most x spikes during the whole contact duration. Right: cumulative percentage of corticostriatal pairs displaying at most x post–pre pairings during contact (for 0 < ∆*t*_STDP_ < 200 ms). Dashed rectangles: typical ranges (5–20 spikes) associated with eCB-LTP in vitro, showing that short-duration contacts have higher probability than long ones to induce eCB-LTP. Black lines: mean; gray area: s.e.m. Two-sided Mann–Whitney *U* test. **c**, The plasticitymeter integrates in vivo spiking data into a corticostriatal STDP model (endocannabinoid- and NMDAR-mediated STDP) to estimate eCB-LTP/LTD events (for eCB-LTD, see Extended Data Fig. 6g). Given the spike times of corticostriatal pairs, the model predicts the occurrence of eCB-LTP and -LTD events. We bin the timing of these events and average across all pairs or all active pairs of corticostriatal neurons to get a density of eCB-LTP events and estimate an instantaneous *Z* score that allows estimating when plasticity is most different from the rest of the recording. Total synaptic weights integrated over pre–post or post–pre pairings ∆*t*_STDP_ are shown as a function of pairing number or 2-AG levels. **d**, Plasticitymeter outputs for all pairs or active pairs for mouse no. 3 aligned with behavior and show a significant *Z* score during contact. Across mice and active pairs, short contacts (mice nos. 1–4) show increased eCB-LTP events compared to longer contacts (mice nos. 5–9). Contacts are indicated (pink bars: <20 s; blue bars >20 s). **e**, Tape avoidance at retrieval (detours) as a function of contact duration at familiarization and cumulative time with eCB-LTP *Z* score >3.59. **f**, Cumulative time with *Z* score >3.59 differs between short (*n* = 4 mice) and long (*n* = 5) contacts (one-sided Mann–Whitney *U* test; *P* = 0.0079). *Z* scores >3.59: events with occurrence probability >99.99%. **g**, In vivo fiber photometry of GRAB_eCB2.0_ in DLS during familiarization shows increased fluorescence at contact onset (average *Z* scores and binned rasters of individual recordings of the fluorescent signal, AUC), absent in GRAB_eCB2.0mut_ or shuffled traces. Two-sided two-sample Student *t*-test (*P* = 0.011). Data: mean ± s.e.m. (**c**,**f**,**g**). *P < 0.05; **P < 0.01; ***P < 0.001 (see ‘Statistics’ in Supplementary Information ‘Methods’).

**Extended Data Fig. 6:**
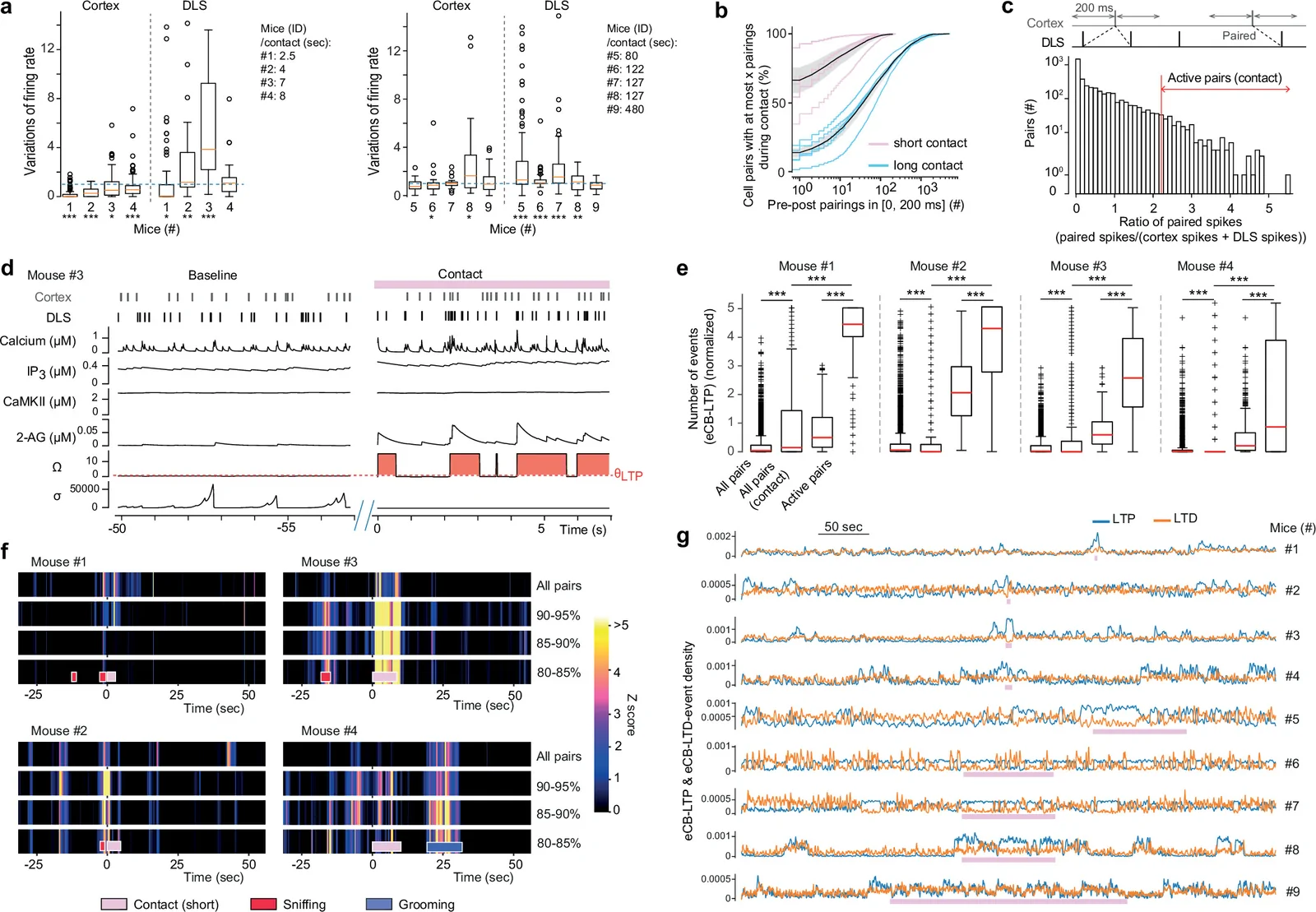
eCB-LTP plasticitymeter. **a**, Boxplots of the ratio of firing rates during contact *vs*. baseline show that the contact induce a reduction in spike number compared to the 95^th^ centile during baseline in most of mice (Mann-Whitney U-test for short contacts in DLS: 1: *U* = 1563, *p* = 0.0169; 2: *U* = 302, *p* = 0.0011; 3: *U* = 30, *p* < 0.001; 4: *U* = 125, *p* = 0.490; in cortex: 1: *U* = 207, *p* < 0.001; 2: *U* = 27, *p* < 0.001; 3: *U* = 1082, *p* = 0.033; 4: *U* = 1124, *p* < 0.001; for long contacts in DLS: 5: *U* = 1781, *p* < 0.001; 6: *U* = 454, *p* < 0.001, 7: *U* = 642, *p* < 0.001, 8: *U* = 326, *p* = 0.0065, 9: *U* = 238, *p* = 0.139; in cortex: 5: *U* = 259, *p* = 0.167; 6: *U* = 136, *p* = 0.016, 7: *U* = 255, *p* = 0.875, 8: *U* = 198, *p* = 0.011, 9: *U* = 1196, *p* = 0.785). Boxplots show the median as the central mark; the box spans from the 25th to the 75th percentile (IQR), and whiskers extend to 1.5×IQR. **b**, Cumulative distribution of cortico-striatal cell pairings during contact (in 0 < ∆t_STDP_ < 200 ms). Black: mean; grey: SEM; colored lines: individual mice with short (pink) or long (blue) contacts (Mann-Whitney U-test: *U* = 0, *p* = 0.0159 for both [0,30] and [0,300] pairing window). **c**, Identification of cortico-striatal pairs with high number of correlated spikes ( ± 200 ms) and during the contact. Active pairs are the top 5% pairs by correlated spikes. **d**, Model simulations of calcium, IP_3_, active CamKII, 2-AG, and endocannabinoid-dependent plasticity variables (Ω and *σ*) for an active pair (mouse #3). eCB-LTP (red) occurs when Ω exceed *θ*_LTP_ and *σ* remains low (*Methods*), arising more frequently during contact. **e**, Rate of eCB-LTP plasticitymeter events (events/second). eCB-LTP events were normalized per cortico-striatal pair to baseline. For each mouse (n = 4), the average plasticitymeter is low when considering all pairs or contact periods (‘all pairs’ or ‘all pairs (contacts)’: ∼4853 pairs per experiment, range 2646-8517). eCB-LTP event rate increased relative to baseline, particularly in active pairs, or pairs more active during contact. Paired two-sample two-sided t-tests showed *p* < 0.001 between conditions for each mouse. Boxplots show the median as the central mark; the box spans from the 25th to the 75th percentile (IQR), and whiskers extend to 1.5×IQR. **f**, Plasticitymeter z-scores across all pairs (top) and active pairs with elevated correlated activity (90-95, 85-90 and 80-85%) during short contacts show that increased eCB-LTP events during contact is robust to the choice of cortico-striatal pair. Pair subsets are selected based on the percentage of increase in correlated activity (90-95, 85-90 and 80-85%) during contact relative to baseline, similarly to the active pairs (corresponding to the 95%-100% range, see panel **c** and Fig. 4d). Z-score maps are aligned to contact onset (time ‘0’). **g**, Distribution of LTP and LTD events across the simulation. In mice experimenting short contacts (#1-4), LTP, but not LTD, induction events increase during contact, preventing possible compensation mechanisms. Across mice, LTD events remain inferior than LTP (1.5-8-fold). Data: mean ± SEM. *p < 0.05; **p < 0.01; ***p < 0.001.

We investigated whether contact with the sticky tape changed population activity level in the cortex and DLS, and whether any such changes could be related to the plasticity expressed in vivo after short contacts (Fig. 4 and Extended Data Fig. 6). To this end, we performed simultaneous in vivo Neuropixels 1.0 recordings in the primary somatosensory cortex and DLS at familiarization (on average per mouse, 58 ± 24 cortical neurons, and 73 ± 39 striatal neurons; *n* = 9 mice) in head-fixed mice inside an air-lifted Mobile HomeCage (MHC), which allows mice to locomote freely despite restraint (Fig. 4a,b and Extended Data Fig. 6a). On average, firing rates were low during contact, with median values ranging between 1 Hz and 3 Hz in both cortex and DLS, even decreasing relative to baseline level in the DLS (Extended Data Fig. 6a). Whereas, on average, 30% of cortical and 27% of striatal neurons were silent during short contacts with the tape (*n* = 4 mice, from 2.5 s to 8 s contact), another 28% of cortical and 23% of striatal neurons exhibited at most 5 to 25 spikes (Fig. 4b). In contrast, more than 90% of neurons fired at least 25 spikes in total during longer contacts (>80 s) with the tape.

We also asked whether correlated firing between pairs of cortical and striatal neurons (here defined as spikes co-occurring within a window of 0–200 ms, that is, a pairing) exhibited changes depending upon whether contacts were short or long. We found that short contacts increased the number of pairs that were not correlated relative to long contacts (61% short versus 11% long), whereas decreasing the number of pairs that were strongly correlated (2% versus 38% neuron pairs with more than 75 pairings). Of note, the percentage of neuron pairs that had between 2 and 25 pairings did not change when comparing short and long contacts (Fig. 4b and Extended Data Fig. 6b). Overall, the sparse firing activity of cortical and DLS neurons during short contacts seems compatible with the specifics of eCB-LTP, elicited in vitro typically by 5–20 pairings at frequencies ranging from 0.1 Hz to 2.5 Hz (ref. ^19^). Based on the above analyses, eCB-LTP is likely to be the dominant form of synaptic plasticity expressed after a short contact, unlike longer contacts (during which more intense and more correlated activity is elicited).

To test this hypothesis, we developed a mathematical model of in vivo corticostriatal STDP that would afford us a quantitative estimate of the amount of endocannabinoid-driven plasticity at each corticostriatal synapse. We took advantage of a validated model^14,19–21,39^ that describes the kinetics of the main molecular species involved in eCB-LTP and eCB-long-term depression (eCB-LTD) signaling pathways as a function of presynaptic and postsynaptic spikes (Fig. 4c). In the model, induction of endocannabinoid plasticity depends on the level of CB1R activation: eCB-LTD is elicited at intermediate endocannabinoid levels and eCB-LTP at high endocannabinoid levels. We used in vivo single-unit cortical and striatal spike trains for each mouse as inputs to the model and estimated the resulting CB1R activation and eCB-LTP induction events, captured by an index called the ‘plasticitymeter’ (Fig. 4c,d and Extended Data Fig. 6d). Active pairs—exhibiting the largest relative increase in correlated activity during contact (Extended Data Fig. 6c)—were associated with high plasticitymeter levels, particularly during behaviorally relevant states: sniffing and contact with the sticky tape, active locomotion and grooming (Fig. 4d and Extended Data Fig. 6e,f). Most eCB-LTP events were concentrated around the contact with the sticky tape; such events were observed in all mice, with plasticitymeter between baseline and contact in active pairs at least doubling for eight out of nine mice.

Short contacts showed an average higher density of eCB-LTP induction events and larger cumulative time of high density eCB-LTP induction events compared to long contacts (mean plasticitymeter values range: 2.2–4.6 versus 0.2–1.5; Mann–Whitney *U* test on the cumulative time: *P* = 0.0079) (Fig. 4d–f). These observations remained consistent when selecting corticostriatal neuron pairs with a smaller increase in their correlated activity during contact, and even in some mice when considering all possible pairs (Extended Data Fig. 6e,f). Synapses spent less time in the eCB-LTD domain, by a factor of 1.5 to 8; in addition, eCB-LTD events were not concentrated during the contact with the tape in short-contact mice (Extended Data Fig. 6g). These modeling results suggest that the increase of eCB-LTP during contact is not counterbalanced by a symmetric increase in eCB-LTD.

### eCB-LTP is engaged specifically in STA after short contacts

Altogether, the plasticitymeter model predicts that the firing patterns of cortical and striatal neurons are compatible with the induction of eCB-LTP specifically during short contacts with the sticky tape. To validate the predictions of the mathematical model, we measured eCB release in vivo during STA with fiber photometry (Fig. 4g). We virally expressed the G protein-coupled receptor-based eCB biosensor (GRAB_eCB2.0_) or its corresponding inactive control (GRAB_eCB2.0mut_)^40,41^ into the DLS and implanted mice with an optic fiber to detect eCB transients at corticostriatal terminals. In STA, we found that eCB levels increased shortly after contact with the tape (lasting about 10 s) in GRAB_eCB2.0_-expressing mice (*n* = 9) relative to randomly time-shifted control traces (two-paired *t*-test; *P* = 0.011) and recordings from GRAB_eCB2.0mut_ mice (*n* = 6) (Mann–Whitney *U* test; *P* = 0.040). These observations nominate eCB signaling as a key mechanism underlying STA-based one-shot learning in the DLS.

**Fig. 5:**
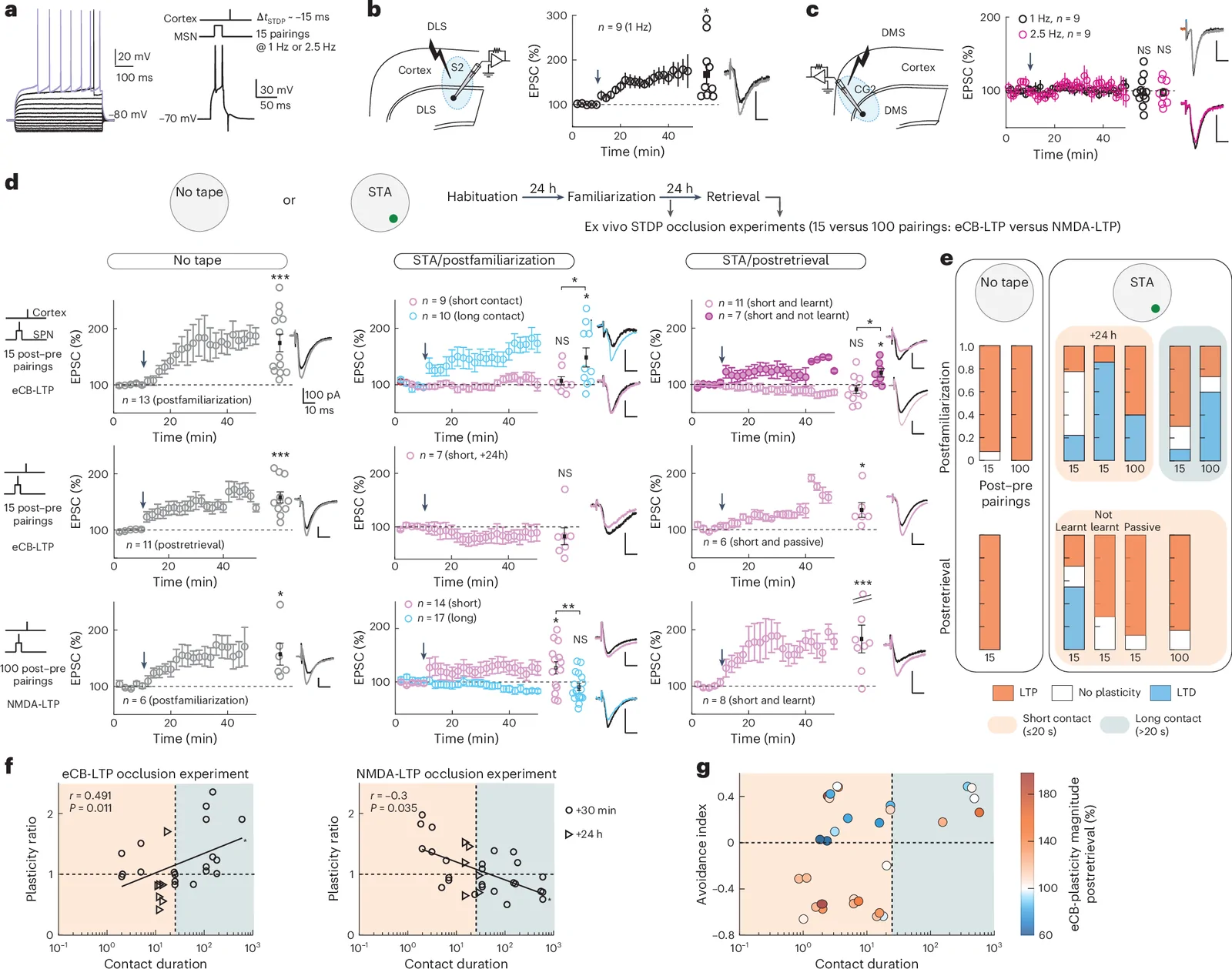
Corticostriatal eCB-LTP is occluded ex vivo after one-shot learning of the STA test, but not after long contact or in nonavoiders. **a**–**c**, eCB-LTP is induced in DLS but not DMS by few corticostriatal pairings. **a**, SPN voltage responses (current pulses from −150 pA to +60 pA above spike threshold, purple trace). STDP protocol: two postsynaptic spikes paired with cortical stimulation (15 pairings, 1 Hz or 2.5 Hz, ∆*t*_STDP_ approximately −15 ms). **b**,**c**, Horizontal (DLS) and parasagittal (DLS) slices; timecourses and EPSC changes (30–40 min after STDP pairings) show LTP in DLS but not DMS (at 1 Hz or 2.5 Hz) (two-sided one-sample Student *t*-test) (**b**) and DMS (**c**) recordings, respectively. Arrow: STDP protocol. **d**–**g**, Ex vivo patch-clamp occlusion experiments in DLS SPNs of mice subjected to STA or no-tape conditions. **d**, STDP protocols: 15 and 100 corticostriatal post–pre pairings at 1 Hz (Δ*t*_STDP_ approximately −15 ms) for eCB-LTP and NMDAR-LTP induction, respectively. In no-tape condition, eCB-LTP (*P* = 0.0005) and NMDAR-LTP (*P* = 0.0276) are induced. In STA, eCB-LTP is occluded following short (*P* = 0.3906) but not long (*P* = 0.0191) contacts. eCB-LTP is occluded in mice showing short contacts during familiarization (≤25 s) and avoidance index >0 (‘short and learnt’) at retrieval (*P* = 0.2409), but preserved in mice with low avoidance index (‘short and not learnt’; *P* = 0.0327) or no active removal (‘short and passive’; *P* = 0.0436). NMDAR-LTP could be induced in short contacts at postfamiliarization (*P* = 0.0391) and at postretrieval (for mice with avoidance index >0, ‘learnt’; *P* = 0.00015) but not in long contacts (*P* = 0.1558). Two-sided one-sample and two-sample independent Student *t*-tests. **e**, Summary of eCB-LTP, NMDAR-LTP and no-plasticity ratios in no-tape and STA conditions after familiarization and retrieval, distinguishing short and long contacts at familiarization. **f**, Inverse correlation between eCB-LTP and NMDAR-LTP magnitude and contact duration at familiarization. Circles and triangles: neurons recorded ex vivo 30 min and 24 h after familiarization, respectively. Pearson correlation two-sided test. **g**, eCB-plasticity at retrieval depends on avoidance index and previous contact duration, with loss of eCB-LTP in high-avoidance mice after short contacts. In **e**–**g**, contact duration ≤25 s in pink and >25 s in blue. All data: mean ± s.e.m. *P < 0.05; **P < 0.01; ***P < 0.001 (see ‘Statistics’ in Supplementary Information ‘Methods’).

To further examine whether the endocannabinoid pathway is engaged in the DLS during STA, we performed ex vivo patch-clamp occlusion experiments in acute brain slices (Fig. 5). We confirmed that eCB-LTP can be elicited in SPNS from the DLS of naive animals upon as few as 15 post–pre pairings^19–22^ (Fig. 5a,b). In contrast, the same induction protocol did not elicit LTP in the dorsomedial part of the striatum (DMS), even when using a higher pairing frequency (2.5 Hz) (Fig. 5c). These results indicate that DLS corticostriatal synapses may be more prone than DMS synapses to express plasticity when few spikes occur.

If eCB-LTP mediates DLS plasticity in vivo after short interactions with the sticky tape, one would predict that this mechanism would be occluded from further plasticity when probed in vitro. To test this hypothesis, we tested eCB-LTP induction in the DLS 30 min or 24 h after familiarization or retrieval in brain slices from mice subjected to the no-tape or STA test (Fig. 5d–g). These two timepoints allow us to disentangle possible short-lasting modulation of neuronal excitability affecting LTP induction from long-term alterations in synaptic strength. In STA, eCB-LTP could not be induced in mice that experienced a short contact with the tape, either 30 min postfamiliarization (*n* = 9 SPNs; *P* = 0.3906) or 24 h later, consistent with eCB-LTP being occluded (*n* = 7 SPNs; *P* = 0.3090; Fig. 5d–f). In contrast, in the no-tape condition, eCB-LTP was induced robustly following 15 pairings, both after familiarization (*n* = 13 SPNs, one-sample *t*-test with *P* < 0.001) and retrieval (*n* = 11 SPNs; *P* < 0.001) (Fig. 5d,e). Furthermore, mice with long contacts still expressed eCB-LTP when tested at +30 min postfamiliarization (*n* = 10 SPNs; *P* = 0.0191).

The observation that eCB-LTP is occluded after short exposure to the sticky tape raises the possibility that the degree of learning observed in vivo might be related to the degree of occlusion observed in vitro. We therefore investigated the relationship between eCB-LTP occlusion and learning, by testing eCB occlusion on short-contact mice whose avoidance index had been quantified after retrieval. We observed an occlusion of eCB-LTP (*n* = 11 SPNs; *P* = 0.2409) in avoidant mice, in contrast to mice that did not learn, that is, exhibiting an avoidance index <0 (*n* = 7 SPNs; *P* = 0.0327; Fig. 5d–f). Note that eCB-LTP could still be induced in mice that did not show avoidance at retrieval and that had removed the tape passively during familiarization (‘short and passive’ group, *n* = 6 SPNs; *P* = 0.0436). These results demonstrate that endocannabinoids are engaged when learning follows a short and active contact with the sticky tape.

In principle, NMDA receptor-mediated plasticity could underlie the changes we observe in the DLS after STA learning. Such pathways have already been implicated in learning-related plasticity expression in vivo^26,27^. We therefore performed occlusion experiments using a paradigm (100 post–pre STDP pairings) known to elicit NMDAR-LTP^19,31^ (Fig. 5d–f). We induced NMDAR-LTP in STA samples (*n* = 14 SPNs; *P* = 0.0391), consistent with this mechanism largely not being occluded by short contacts to the sticky tape; note that this result is the opposite of what we observed when querying occlusion of eCB-LTP. NMDAR-LTP could also be induced reliably postretrieval in one-shot learners (*n* = 8 SPNs; *P* = 0.00015). In contrast, NMDAR-LTP was occluded in mice who had long-duration contacts with the tape (*n* = 17 SPNs; *P* = 0.1558). NMDAR-LTP was also induced readily in the no-tape condition (*n* = 6 SPNs; *P* = 0.0276).

Together, these findings indicate that the endocannabinoid system, rather than NMDAR signaling, is engaged in the DLS immediately following a one-shot experience, supporting one-shot learning and likely underlying corticostriatal potentiation of ChR2-LFP.

### Impaired one-shot learning in eCB-LTP knockout mice

**Fig. 6:**
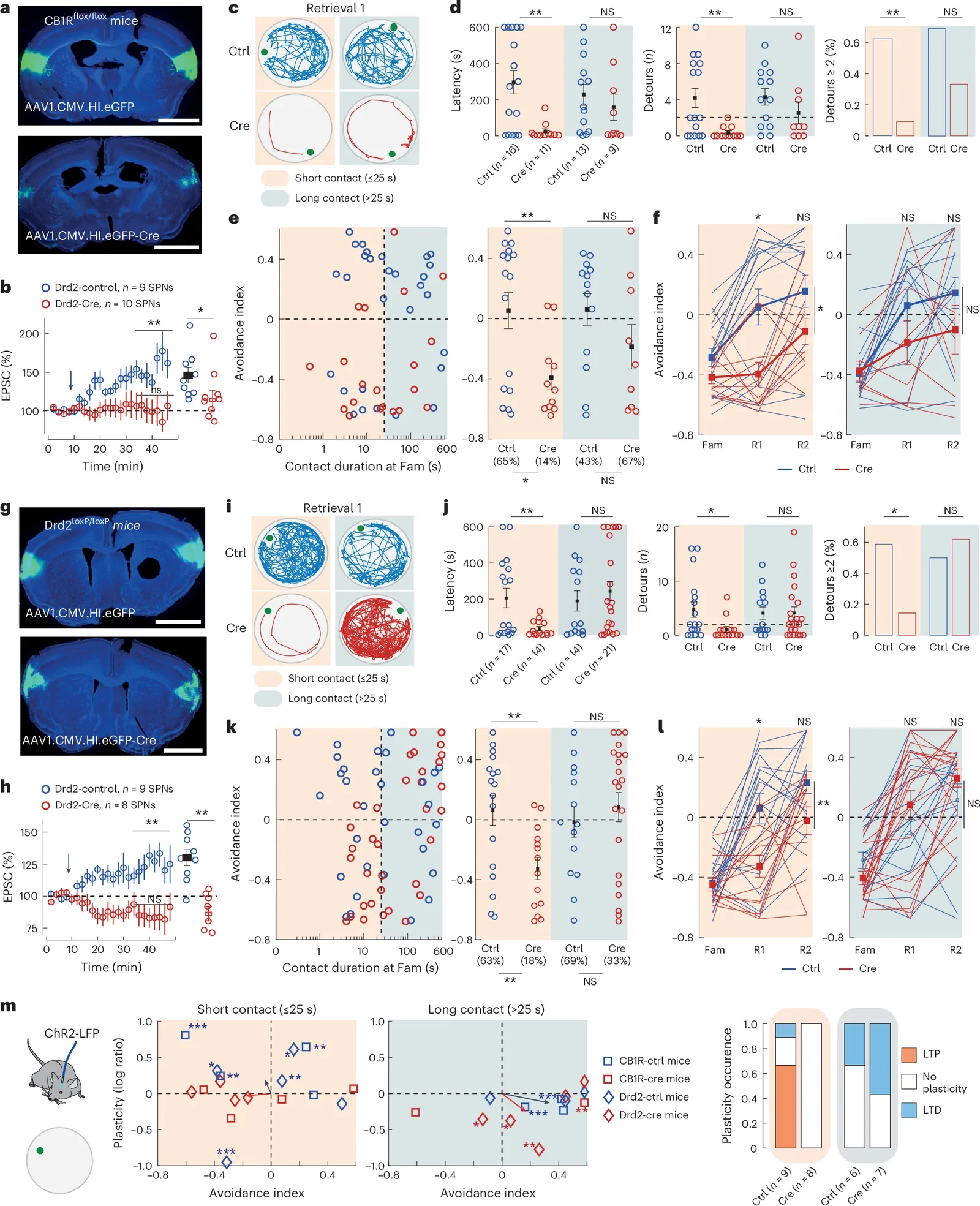
One-shot learning of the STA test is impaired in corticostriatal presynaptic CB1R or D2R conditional knockout mice. **a**,**g**, Bilateral virus expression (AAV1.CMV.HI.eGFP for control (Ctrl) or AAV1.CMV.HI.eGFP-Cre (Cre) for mice in which CB1R or D2R are deleted) targeting somatosensory S2 cortex in CB1R^flox/flox^ (**a**) and Drd2^LoxP/LoxP^ (**g**) mice. Scale bars, 0.5 mm. **b**,**h**, Ex vivo whole-cell recordings show impaired eCB-LTP after STDP (15 post–pre pairings, 1 Hz, Δ*t*_STDP_ approximately –15 ms) in conditional knockouts. Timecourses and EPSC changes (scatterplots, 30 min after STDP pairings). Two-sided one-sample and two-sample Wilcoxon test. **c**,**i**, Illustrative trajectories at retrieval: control mice avoid the tape after short contact, whereas knockouts do not; both perform equally well for long contacts. **d**,**j**, Latency-to-contact, detours and proportion of mice with at least two detours. Two-sample Student *t*-tests (short contact: ≤25 s). **e**,**k**, Avoidance index and avoiders (percentage indicated at the bottom) are low in knockout mice after short contact at familiarization, but remain high for long contact. Two-sided two-sample Student *t*-tests, Chi-squared test. In CB1R^flox/flox^: avoidance index for short contacts: *P* = 0.0094; long contacts: *P* = 0.174; avoider rate for short contacts: *P* = 0.022; long contacts: *P* = 0.198; in Drd2^LoxP/LoxP^: avoidance index for short contacts: *P* = 0.005; long contacts: *P* = 0.498; avoider rate for short contacts: *P* = 0.004; long contacts: *P* = 0.163. **f**,**l**, Avoidance index across sessions (familiarization, retrieval-1 and -2) for short and long contact. Linear mixed models with post hoc two-sided Student *t*-tests (Tukey correction). Circles and thin lines: individual mice. **m**, ChR2-LFP monitoring in CB1R^flox/flox^ and Drd2^LoxP/LoxP^ mice. Plasticity log ratio at familiarization +24 h as a function of STA avoidance index at retrieval for short contacts (≤25 s, left) or long contacts (middle). Control mice show more potentiation than knockouts for short contacts, whereas long contacts yield similar LTD/no plasticity distributions (right). Two-sided Fisher’s exact test. All data: mean ± s.e.m. *P < 0.05; **P < 0.01; ***P < 0.001 (see ‘Statistics’ in Supplementary Information ‘Methods’).

To investigate whether eCB-LTP is required for one-shot learning, we took advantage of the fact that presynaptic activation of CB1R and dopaminergic type-2 receptors (D2R) is necessary for eCB-LTP expression in vitro^20^. We generated conditional eCB-LTP knockouts by expressing Cre recombinase virally in the secondary somatosensory S2 cortex of CB1R^flox/flox^ or Drd2^LoxP/LoxP^ mice (CB1R-deleted or Drd2-deleted mice) with green fluorescent protein (GFP)-expressing mice serving as controls (CB1R-control or Drd2-control mice; Fig. 6a,g). We confirmed that eCB-LTP was impaired in CB1R-deleted and Drd2-deleted mice (Fig. 6b,h): in acute slices from naive mice, the 15 post–pre corticostriatal STDP pairings led to eCB-LTP expression in CB1R-control and Drd2-control mice (*n* = 9 and *n* = 9 SPNs, respectively), but not in CB1R-deleted and Drd2-deleted mice (*n* = 10 and *n* = 8 SPNs, respectively). Hence, we hypothesized that one-shot learning should be impaired in eCB-LTP knockout mice and assessed the behavior of CB1R and D2R conditional knockouts during STA (*n* = 20 CB1R-deleted, *n* = 29 CB1R-control mice; *n* = 35 Drd2-deleted, *n* = 31 Drd2-control mice).

**Extended Data Fig. 7:**
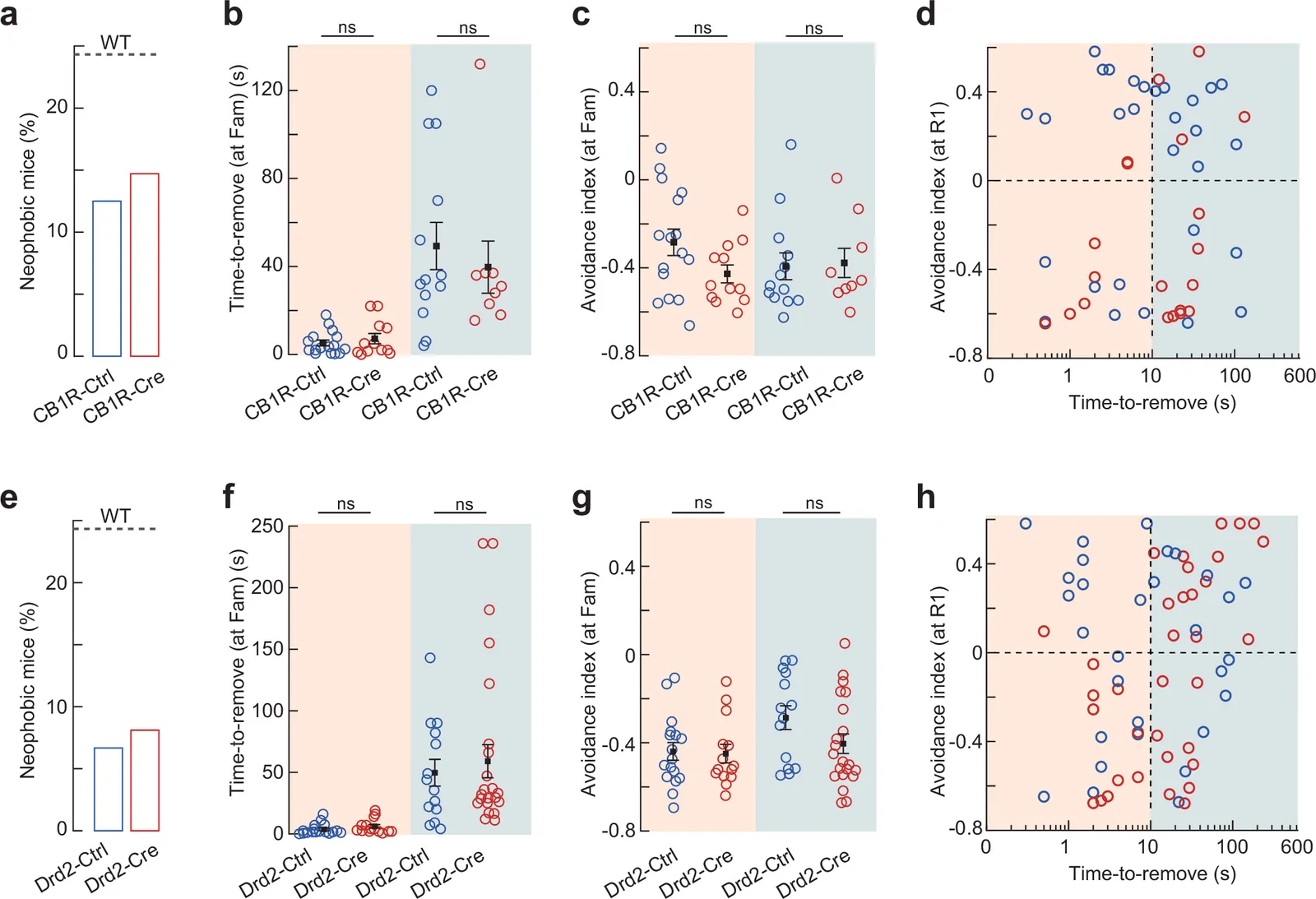
Behavioral characterization of eCB-LTP conditional knockout mice for presynaptic CB1 or D2 receptor. **a**,**e**, Percentage of mice exhibiting avoidance at familiarization, that were excluded from the rest of our analyses in CB1R^flox/flox^ mice (**a**) or Drd2^*LoxP/LoxP*^ (**e**), in comparison to the percentage found in control wild-type mice (dashed line). **b,f**, No significant differences in time-to-remove were observed in CB1R^flox/flox^ mice (**b**, n = 25 CB1R-deleted and n = 29 CB1R-control mice) or Drd2^*LoxP/LoxP*^ (**f**, n = 35 Drd2-deleted and n = 31 Drd2-control mice)**)**. Two-sample two-sided independent t-tests for CB1R^flox/flox^ mice (short: *t* = -0.568, *p* = 0.575; long: t = 0.590, *p* = 0.562) and *Drd2*^*LoxP/LoxP*^ (short: *t* = -1.331, *p* = 0.193; long: *t* = -0.496, *p* = 0.623). **c,g**, Comparison of avoidance index at familiarization for CB1R^flox/flox^ mice (**c**) or Drd2^*LoxP/LoxP*^ (**g**) does not reveal significant differences in the first interaction with the sticky tape. Two-sample two-sided independent t-tests for CB1R^flox/flox^ mice (short: *t* = -0.035, *p* = 0.115; long: *t* = -0.207, *p* = 0.868) and Drd2^*LoxP/LoxP*^ (short: *t* = 0.178, *p* = 0.860; long: *t* = 1.670, *p* = 0.100). **d,h**, Avoidance index at retrieval-1 as a function of the time-to-remove at familiarization in CB1R^flox/flox^ mice (**d**) or Drd2^*LoxP/LoxP*^ (**h)**. All data: mean ± SEM. *p < 0.05; **p < 0.01; ***p < 0.001.

At familiarization, all mice behaved similarly regardless of genotype (Extended Data Fig. 7a–c,e–g). In contrast, at retrieval, CB1R-deleted mice showed impaired one-shot learning after short contact. Such mice exhibited reduced latency-to-contact and fewer detours near the tape compared to controls (two-sample *t*-tests, latency-to-contact: *P* = 0.006; detours: *P* = 0.0215; *n* = 11 versus 16) (Fig. 6c,d). Only a small fraction of short-contact CB1R-deleted mice avoided the tape (18% avoiders), whereas CB1R-control mice performed similarly to wild-type animals with 63% of avoiders (avoidance index: two-sample *t*-test, *P* = 0.0094; avoider rate: *χ*^2^-test; *P* = 0.022; Fig. 6e and Extended Data Fig. 7d). Similarly, short-contact Drd2-deleted mice displayed impaired one-shot learning. At retrieval, they showed reduced latency-to-contact and fewer detours relative to Drd2-control mice (two-sample *t*-tests, latency-to-contact: *P* = 0.0078; detours: *P* = 0.030; *n* = 14 versus 17; Fig. 6i,j). Their avoidance index and avoider rate were markedly lower than in controls (avoidance index: two-sample *t*-test, *P* = 0.006; avoider rate: *χ*^2^-test, *P* = 0.018; Fig. 6k and Extended Data Fig. 7h). In contrast, the behavior of long-contact CB1R-deleted mice (*n* = 9) was not significantly different from CB1R-control animals (*n* = 13) (avoidance index: two-sample *t*-test; *P* = 0.174; avoider rate: *χ*^2^-test; *P* = 0.198). Similarly, long-contact Drd2-deleted mice (*n* = 21) exhibited robust avoidance behavior comparable to that of Drd2-control mice (*n* = 14) (avoidance index: two-sample *t*-test, *P* = 0.498; avoider rate: *χ*^2^-test; *P* = 0.163).

Overall, these data show that one-shot learning is prevented when eCB-LTP is impaired, specifically in animals experiencing a short contact duration with the sticky tape. When mice underwent two retrieval sessions 24 h apart, knockout and control mice exhibited distinct behaviors for short contact duration during the first retrieval session, whereas no difference was observed at the second retrieval (Figs. 6f,l). Thus, the endocannabinoid and dopaminergic systems, acting at cortico-DLS synapses through CB1R and D2R at presynaptic cortical afferents, are critical for one-shot learning but not for forming memories after a second iteration of the same experience.

These results suggest that presynaptic deletion of CB1R and/or D2R prevents striatal plasticity. To validate this hypothesis, we monitored in vivo ChR2-LFP in CB1R and Drd2 conditional knockout mice during the STA test. We found that, among short contacts, LTP of ChR2-LFP was expressed in 67% of control mice (*n* = 9) versus 0% in knockout mice (*n* = 8) (Fisher’s exact test; *P* = 0.009; Fig. 6m). As in wild-type mice (Fig. 3), long contacts did not elicit any potentiation of ChR2-LFP in both control (*n* = 6) and knockout mice (*n* = 7) but instead a mixed occurrence of LTD and no plasticity (33% versus 57% LTD, respectively, Fisher’s exact test; *P* = 0.592; Fig. 6m). However, unlike wild-type mice, potentiation of ChR2-LFP after short contact was not restricted to avoidant mice in the control group (that is, two nonavoiders also displayed LTP of ChR2-LFP whereas two avoiders did not display potentiation).

**Extended Data Fig. 8:**
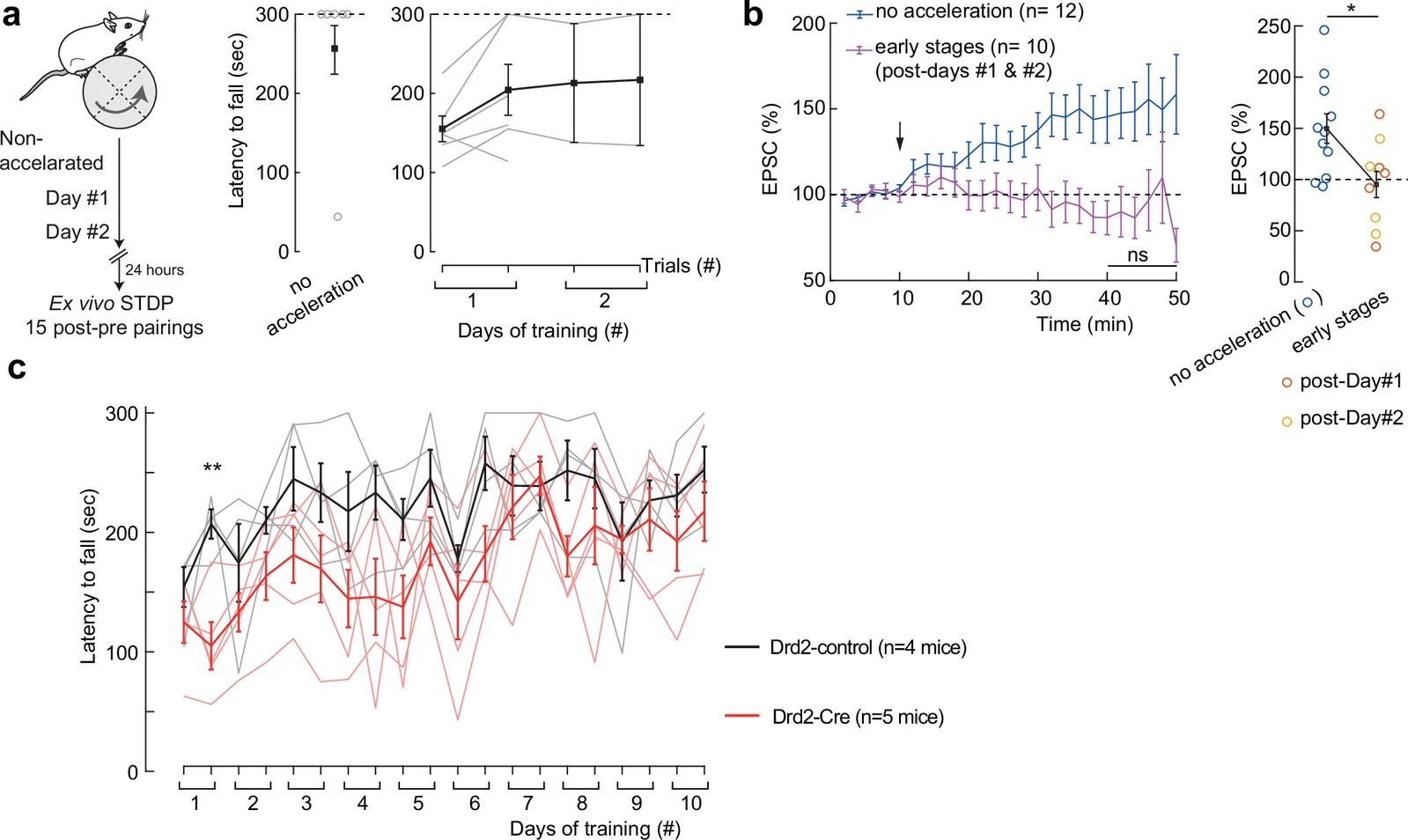
eCB-LTP is engaged in the early stages of the accelerating rotarod. **a**, Experimental set-up: Rotarod and *ex vivo* STDP in brain slices. Performance of wild-type mice on the non-accelerating (control mice, n = 6) and accelerating rotarod (2 sessions per day; n = 6). Motor skill learning of mice is evaluated with the time spent on the rotarod before falling. **b**, Average time-courses of patch-clamp *ex vivo* STDP occlusion experiments performed 30 min post-rotarod in SPNs in the DLS; the arrow indicates the time of the eCB-LTP 15 STDP pairing protocol (Δt_STDP_ ~ -15ms). Scatter plots represent the percentage of change in EPSC amplitude 30-40 min after STDP pairings. While SPNs from control mice (non-accelerating rotarod, n = 12 SPNs) can still induce eCB-LTP (one-sample two-sided Student t-test: *t* = 3.47, *p* = 0.0061), eCB-LTP is occluded after both 2 or 4 training sessions in the accelerating rotarod (n = 10 SPNs) (*t* = -0.384, *p* = 0.709), leading to a significant difference between the early stages *vs*. control group (two-sample Student t-test: *t* = 2.82, *p* = 0.0108). **c**, Comparison of the performance of Drd2^*LoxP/Lox*^ mice on the accelerating rotarod shows a significant difference between the Drd2-deleted (n = 5 mice) and Drd2-control (n = 4) groups (Repeated-Measure ANOVA, Group Effect: *F* = 6.75, *p* = 0.0354) with a significant impairment on the second session of day #1 in Drd2-cre (post hoc two-sided two-sample t-tests: on day #1-session 2: *t* = 4.081, *p* = 0.0046 *vs*. on day #1-session 1: *t* = 1.197, *p* = 0.270). All data: mean ± SEM. *p < 0.05; **p < 0.01; ***p < 0.001.

The mechanisms identified here could in principle support initial learning phases in various settings. We therefore investigated whether eCB-LTP contributes to the initial learning stages of procedural learning. Indeed, cortico-DLS plasticity is a key element in procedural learning, which typically comprises rapid initial learning followed by a slower phase^26,28,42^. We assessed motor learning using an accelerating rotarod paradigm with limited training (two trials per day for ten consecutive days) to prolong the early acquisition stage (Extended Data Fig. 8a). Ex vivo occlusion experiments performed after the first or second day showed an occlusion of eCB-LTP in mice subjected to the accelerating rotarod (*n* = 10 SPNs, one-sample *t*-test, *P* = 0.709), unlike control mice subjected to a single session of the nonaccelerating rotarod (*n* = 12 SPNs, two-sample *t*-test, *P* = 0.0108; Extended Data Fig. 8b). We further observed that Drd2-deleted mice (*n* = 5) showed decreased behavioral performance during the first 2 days compared to Drd2-control mice (*n* = 4; Extended Data Fig. 8c). These results indicate that eCB-LTP could also be engaged in the initial trials of motor skill learning.

### Inhibition of CB1R, but not NMDAR, impairs one-shot learning

**Fig. 7:**
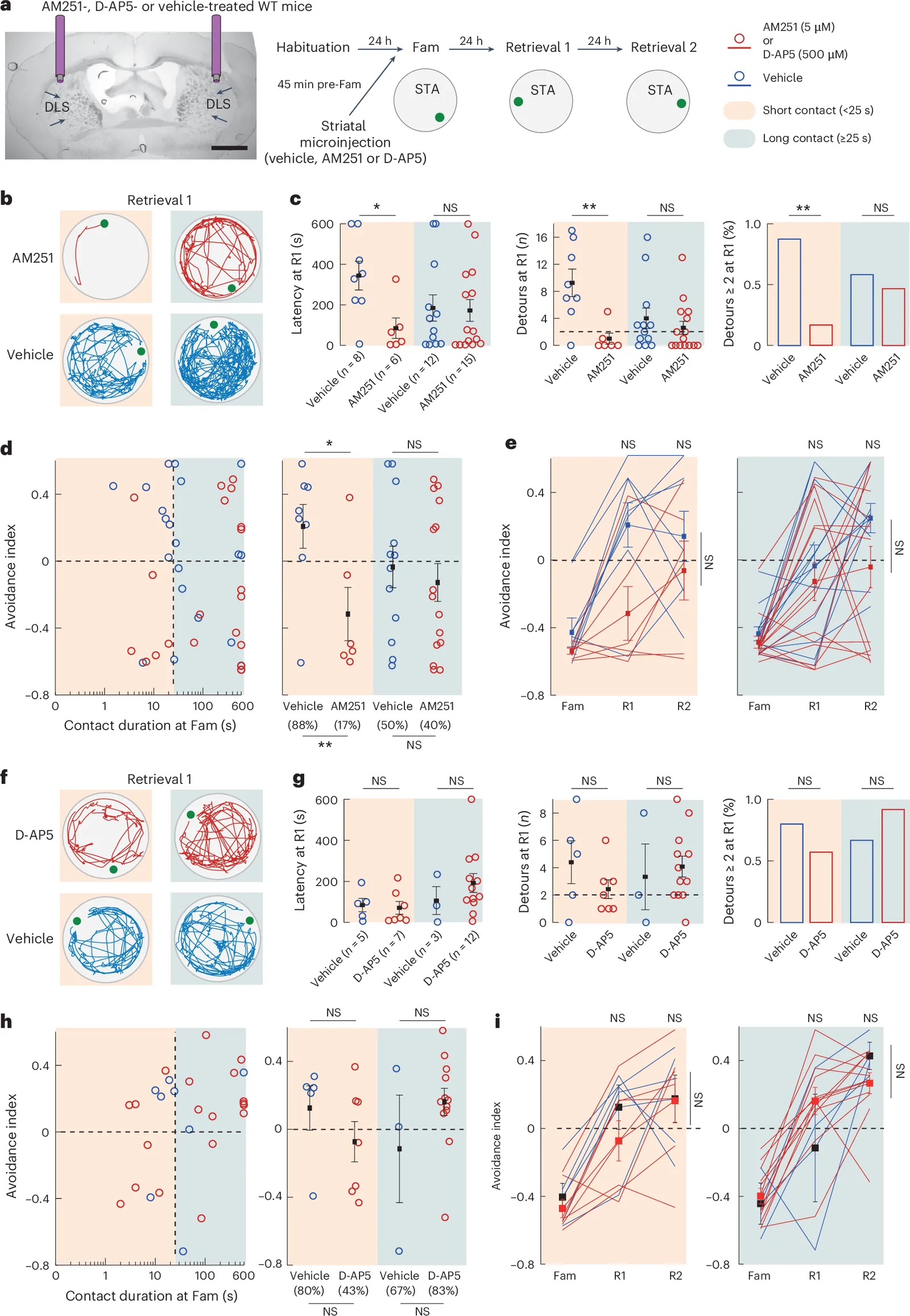
Transient inhibition of CB1R, but not NMDAR, impairs one-shot learning. **a**–**i**, Wild-type mice were injected bilaterally (**a**), using cannulas inserted into two guide cannulas implanted in each DLS (arrows: injection sites; scale bar, 0.25 mm) with vehicle or AM251 (5 μM) (**b**–**e**) or D-AP5 (500 μM) (**f**–**i**) solutions 45 min before STA familiarization (Fam). **b**–**e**, One-shot learning was impaired in AM251-injected mice. **b**, Representative retrieval-1 trajectories of AM251- or vehicle-injected mice for short and long contacts (at familiarization). Latency-to-contact, detours and proportion of mice showing at least two detours (**c**), avoidance index (circles) and avoiders (%) at retrieval-1 (**d**), timecourses of the avoidance index at familiarization, and retrieval-1 and retrieval-2 (**e**) indicate impairment of one-shot learning in AM251-injected mice with short, but not long, contact versus vehicle-injected mice (avoidance index for short contacts: *P* = 0.0250; long contacts: *P* = 0.587; avoider rate for short contacts: *P* = 0.00804; long contacts: *P* = 0.6033). **f**–**i**, One-shot learning was not affected in D-AP5-injected mice. **f**, Representative retrieval-1 trajectories of D-AP5- or saline-injected mice for short and long contacts (at familiarization). Latency-to-contact, detours and proportion of detours ≥2 (**g**), avoidance index and avoiders (**h**) show no difference for short and long-contact duration in D-AP5- and saline-injected mice (avoidance index for short contacts: *P* = 0.292; long contacts: *P* = 0.219; avoider rate for short contacts: *P* = 0.198; long contacts: *P* = 0.519). **i**, D-AP5- and saline-injected mice, with a short contact duration at familiarization, display similar time course of the avoidance index at familiarization, retrieval-1 and retrieval-2. Two-sided two-sample Student *t*-tests (**c**,**d**,**g**,**h**), Chi-squared test (**c**,**d**,**g**,**h**). Linear mixed model with Tukey correction applied to post hoc two-sided *t*-tests (**e** and **i**). Circles and thin lines: individual mice. All data: mean ± s.e.m. *P < 0.05; **P < 0.01; ***P < 0.001 (see ‘Statistics’ in Supplementary Information ‘Methods’).

**Extended Data Fig. 9:**
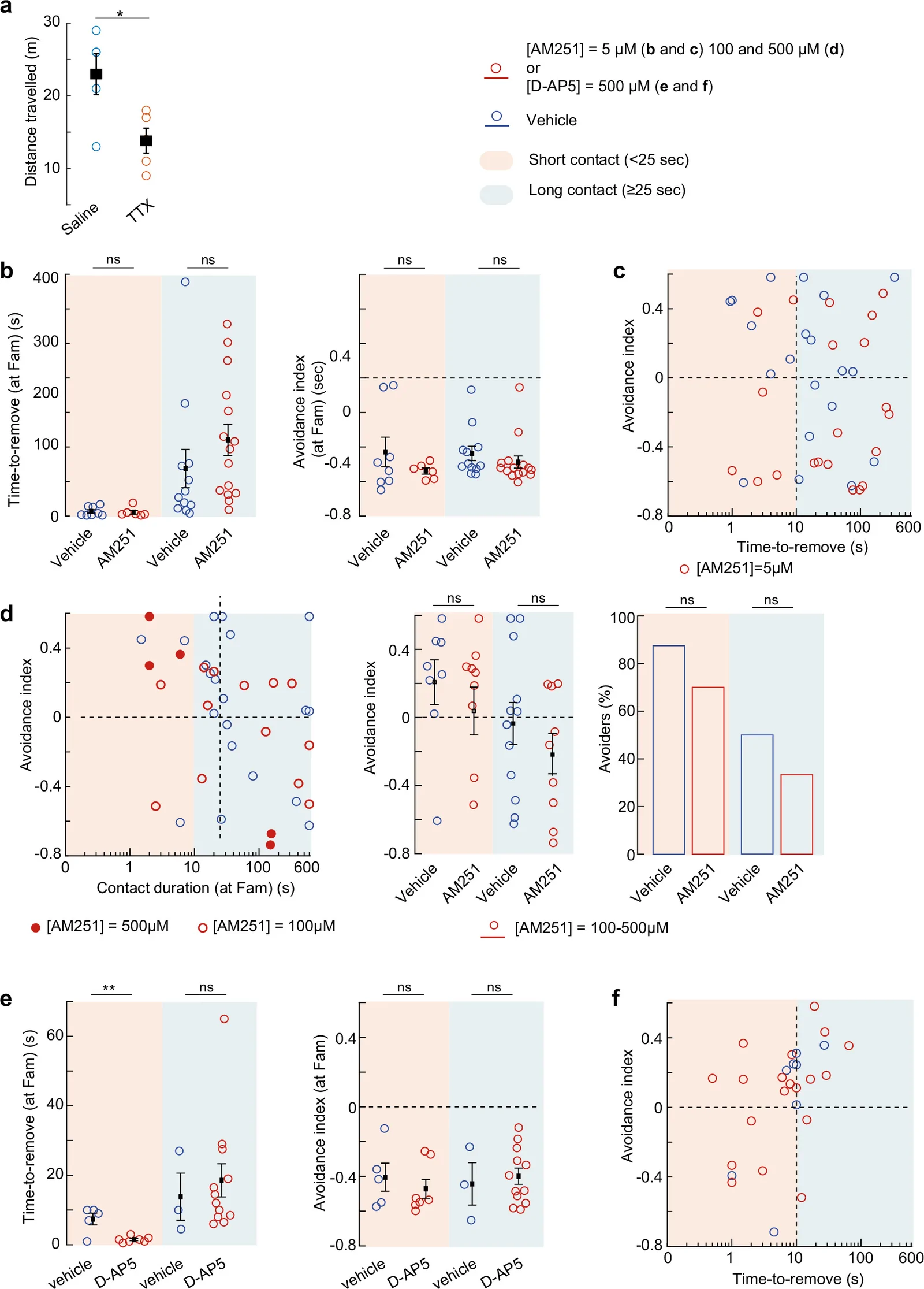
Impact of AM251 and D-AP5 on behavioral performance. **a**, Efficiency of drug-injected mouse *via* bilateral cannulas. The distance travelled by mice injected with TTX (1 µM, 1-2 µL, n = 4 mice) was significantly lower than for mice injected with saline (2 µL, n = 4 mice)) 45 min before open field test. Two-sample two-sided independent t-test: *t* = 2.79; *p* = 0.023. **b**, Absence of impact of AM251 (5 µM) at familiarization on time-to-remove and avoidance index. Two-sample two-sided independent t-tests on time-to-remove for short contacts: *t* = 0.326, *p* = 0.750 and long contacts: *t* = -1.171, *p* = 0.253; on avoidance index at familiarization for short contacts: *t* = 1.088, *p* = 0.298 and long contacts: *t* = 0.930, *p* = 0.361. **c**, Avoidance index at retrieval as a function of the time-to-remove the sticky tape (at familiarization) shows low values for AM251-injected mice for active removal <10 seconds, in contrast to vehicle-injected mice. **d**, High concentration of AM251 (100 to 500 µM) does not induce one-shot learning impairment in drug-injected mice. Avoidance index at retrieval as a function of the contact duration at familiarization. Summary scatter plot of the avoidance index shows no significant differences (two-sample two-sided independent t-tests) between short contacts (≤25 seconds; *t* = 0.864, *p* = 0.400, n = 9 AM251-injected *vs*. n = 8 vehicle-injected) and between long contacts (*t* = 1.020; *p* = 0.320, n = 9 AM251-injected *vs*. n = 12 vehicle-injected). Similarly, the percentage of avoiders does not vary across conditions (χ^2^-tests: for short contacts χ^2^ = 0.788, p = 0.375; for long contacts χ^2^ = 0.583, p = 0.445). **e**, Impact of D-AP5 (500 µM) at familiarization on time-to-remove and avoidance index. Two-sample two-sided independent t-tests on time-to-remove for short contacts: *t* = 4.073, *p* = 0.0022 (n = 7 D-AP5-injected *vs*. n = 5 vehicle-injected) and long contacts: *t* = -0.487*, p* = 0.626 (n = 12 D-AP5-injected *vs*. n = 3 vehicle-injected); on avoidance index at familiarization for short contacts: *t* = 0.716, *p* = 0.490 and long contacts: *t* = -0.404, *p* = 0.693. **f**, Avoidance index as a function of the time-to-remove (at familiarization) shows an absence of effect of D-AP5 infusion on avoidance performance. All data: mean ± SEM. *p < 0.05; **p < 0.01; ***p < 0.001.

To determine whether a transient blockade of CB1R impairs one-shot learning rather than memory expression/retrieval, we inhibited CB1R immediately before familiarization. For this purpose, we inhibited CB1R pharmacologically in the DLS 30 min before familiarization using bilateral AM251 (5 μM) microinfusions^43^ using implanted guide cannulas; vehicle (DMSO 0.5–10% in saline) microinfusions served as controls (*n* = 21 AM251-injected mice and 20 vehicle-injected mice) (Fig. 7a–e and Extended Data Fig. 9a). During familiarization, all vehicle- and AM251-injected mice showed similar behavior (Extended Data Fig. 9b). AM251-injected mice with a short contact at familiarization showed reduced latency-to-contact and fewer detours at retrieval, compared to vehicle-injected mice (independent *t*-tests, latency-to-contact: *P* = 0.017; detours: *P* = 0.006; detours ≥2: *P* = 0.008; *n* = 6 versus *n* = 8) (Fig. 7b,c), and a lower avoidance index and avoider rate (avoidance index: independent *t*-test, *P* = 0.025; avoider rate: *χ*^2^-test; *P* = 0.008; Fig. 7d,e and Extended Data Fig. 9c). In contrast, vehicle- and AM251-injected mice with long contact at familiarization, displayed similar behavior at retrieval (Fig. 7b–e). When tested on a second session of retrieval, both groups behaved similarly, regardless of their contact duration at familiarization (Fig. 7e). Note that, in accordance with AM251 dose-response efficiency^44^, no effect on one-shot learning was observed for higher concentrations (100 μM or 500 μM; Extended Data Fig. 9d).

To further examine the contribution of NMDAR-mediated signaling in STA, especially in the long-contact condition in which NMDAR-LTP was, on average, occluded ex vivo, we inhibited NMDAR pharmacologically in the DLS 30 min before familiarization (bilateral D-AP5 500 μM; *n* = 19 mice); saline microinfusions served as controls (*n* = 8 mice; Fig. 7a and Extended Data Fig. 9a). At familiarization, D-AP5- and saline-injected mice exhibited similar behaviors (Extended Data Fig. 9e). At retrieval, we did not observe significant differences in D-AP5- and saline-injected mice in the latency-to-contact, number of detours, proportion of detours ≥2, avoidance index and avoider rate, regardless of contact duration at familiarization (Fig. 7f–I and Extended Data Fig. 9f). Avoidance index was also unchanged in both conditions on the second retrieval session. Thus, transient manipulation in the DLS of NMDA signaling does not influence one-shot learning in vivo.

## Discussion

The STA test induces long-term memory in almost two out of three mice after a one-shot experience, which persists for at least 1 month. Notably, avoidance learning in the STA test does not depend on the duration of contact or the cumulative time spent attempting to remove the tape. Although the sticky tape is a surprising and mildly uncomfortable stimulus that leads most mice to avoid it on the next exposure, it does not induce fear-related behaviors or repeated stress, as indicated by the careful but repeated investigations of the tape, and by corticosterone measurements. The STA test also elicits a rich repertoire of spontaneous behaviors as reflected in the heterogeneity of contact, tape removal attempts and avoidance strategies. Hence, this new behavioral test expands the spectrum of one-shot learning behaviors, which have been restricted largely to spatial learning^45^ or to fear-based paradigms^46^. STA test further opens up the opportunity to examine the contributions of the basal ganglia, which has thus far been understudied as a site of one-shot learning relative to hippocampal and amygdalar circuits.

Our results provide insights into the temporal organization of synaptic plasticity underlying learning from a single experience. Previous in vitro work examining the sensitivity of corticostriatal synapses to undergo plasticity following brief versus more intense synaptic stimulation demonstrated the activation of two distinct pathways: eCB-LTP versus NMDAR-LTP^19^. In the same vein, we found that brief sticky tape contacts relied on and required eCB-LTP but not NMDAR-LTP, unlike longer contacts. Moreover, beyond its low threshold for induction, eCB-LTP is more robust to spike-timing jitter than NMDAR-LTP^21^—a feature that may further bias the reliance of one-shot learning on endocannabinoids rather than NMDAR signaling, as reduced spike-timing variability typically emerges at later learning stages^32,47^. Finally, eCB-LTP is sensitive to neuromodulators, such as dopamine, which encodes the saliency or value of cortical inputs^48^ and can rapidly modulate striatal neuron activity in response to threats^49^. Dopamine transients in the nucleus accumbens, driving active avoidance, also depend on endocannabinoid release from the ventral tegmental area^50^, leaving open questions regarding their respective interactions in STA learning. Likewise, it remains to be determined whether glucocorticoids, which enhance memory and promote avoidance through CB1R activation in the DLS^51^, modulate the threshold for eCB-LTP induction. Furthermore, the critical involvement of endocannabinoids, and not of NMDAR, in one-shot learning, aligns with studies reporting impaired learning in inhibitory avoidance tasks upon CB1R—but not NMDAR—inhibition after a single training session^51,52^, and the absence of change in AMPA/NMDA ratio in DLS together with no learning deficits after NMDAR blockade during the initial trials of the rotarod^26,29^. Our results, although preliminary, suggest that the initial stages of motor learning in the rotarod also depend upon eCB-LTP and cortical D2R. The mechanisms identified for STA learning could therefore generalize to other settings. Future work should test the role of eCB-LTP in early learning across tasks spanning supervised and unsupervised paradigms, as well as continuous and discrete (repeated discrete epochs) learning regimes.

Although converging evidence supports DLS eCB-LTP as a substrate of one-shot learning in the STA test, the mechanisms engaged after prolonged contact require further work. Indeed, after a long contact, no LTP was detected in vivo in the DLS; ex vivo induction of eCB-LTP was not affected; and neither conditional CB1R and D2R knockouts nor pharmacological inhibition of CB1R affected learning. Partial occlusion of NMDAR pathway ex vivo did not translate into detectable corticostriatal LTP of ChR2-LFP in vivo, suggesting that LTP might be restricted to a subset of synapses, or that additional mechanisms (such as eCB-LTD) may mask NMDAR-LTP signatures in vivo. In addition, contrary to our initial hypothesis, pharmacological inhibition of NMDARs did not impair learning following long contacts. Altogether, these results indicate that other brain circuits probably support avoidance behavior when the initial experience is not brief. Whether DMS or cortical circuits override the function of DLS remains to be determined.

Upon second exposure to the tape, mice lacking eCB-LTP no longer show impaired learning, indicating that consolidation and memory transfer proceed independently of the eCB-LTP expression. These findings support a role for eCB-LTP as a priming mechanism, triggered by salient and brief stimulation and initially recruiting a broad neuronal population, consistent with in vivo long-term synaptic changes and widespread ex vivo occlusion of eCB-LTP. This priming may be followed by refinement and consolidation of plasticity at corticostriatal synapses and in distributed brain circuits, leading to the selection and stabilization of specific subsets of cells, in line with previous reports of evolving striatal activity patterns across learning trials^26,29–31,53^. Future studies could test the stability of LTP expression in the DLS in vivo as well as the dynamics of eCB versus NMDAR-LTP occlusion ex vivo, over time and across repeated exposures to the STA test. Finally, rapid inter-regional transfer of memory traces should not be excluded, as suggested by lesion studies showing a shift from hippocampal to DLS dependence between first and second trials^52^. Overall, our work highlights that distinct synaptic substrates underlie learning after single brief versus long experiences and one- versus two-shot learning.

Although DLS is described as being engaged in later learning phases associated with habit formation^28,54^, mounting evidence indicates DLS involvement during early training phases^29–31,54^. DLS is also involved in fast behavioral dynamics, as it encodes subsecond behavior syllables^55^ and integrates rapid dopamine transients^56^. Furthermore, we did not observe eCB-LTP in vitro in DMS, classically engaged in early learning phases and rapid adaptations to new action-outcome associations^26,31,34^. Such differential sensitivity to sparse firing activation could add another layer of functional segregation between these circuits, which exhibit distinct plasticity rules^31^, and underscore the critical role of the DLS in mediating one-shot learning upon a very brief experience. Future work should investigate whether additional one-shot synaptic plasticity rules, relying on dendritic calcium plateau potentials in SPNs^57,58^ and akin to BTSP^3,15,16^, also occur in striatum.

BTSP has been proposed as a one-shot learning rule for tuning hippocampal pyramidal cells to a variety of features (place fields, association of reward and stimuli) while linking temporally distant events (>1 s)^3^, suggesting a general role in rapid memory formation^59^. In contrast, STDP has been considered limited to fast integration timescale (tens of milliseconds) and vulnerable to noise^3^, leading to the proposal of a speed-amplitude trade-off between STDP and BTSP governing plasticity^3,59^. However, evidence indicates that STDP can integrate temporally distant events through long eligibility traces^60^, even at low frequencies (0.1 Hz)^11,61,62^ and support rapid associative learning^63^. In the DLS, eCB-LTP is robust to noise and can be induced at 0.1 Hz^19,21^. Depending on SPNs activity regimes, cortical inputs and neuromodulation, BTSP and/or STDP may support one-shot learning.

Finally, eCB-LTP is an atypical plasticity mechanism and its feature and generalizability warrant additional work. Although endocannabinoids have long been linked to learning and memory^64^, their role is attributed to synaptic depression, with eCB-LTD implicated in fear memory^65^, suppression of instinctive fear^66^ or alcohol-seeking behaviors^67^. However, accumulating evidence indicates that endocannabinoids can also enhance neurotransmitter release^22,63^, for example at cortical layer 2/3–layer 5 synapses with similar mechanisms^68^, or in dentate gyrus following a high-frequency stimulation^69^. Together, our findings expand the role of the DLS in learning and highlight the bidirectional nature of endocannabinoid-mediated plasticity as a key framework for understanding its functions in learning.

## Methods

### Animals

Experiments were performed in accordance with local animal welfare committee (CIRB) and EU guidelines (directive 2010/63/EU). P7–10 weeks wild-type C57BL6/J mice (Charles River), Drd2^loxP/loxP^ mice (JAX stock no. 020631) and CB1R^flox/flox^ mice (gift from G. Marsicano’s laboratory, Bordeaux, France) of both sexes were group-housed on a standard 12-h light/dark cycle and with ad libitum food and water. Homozygous Drd2^loxP/loxP^ and CB1R^flox/flox^ were obtained from heterozygous breeding mice (back-crossed with C57BL6/J mice).

For surgeries, mice were anesthetized intraperitoneally with a mix of ketamine (67 mg kg^−1^, Imalgène, Mérial) and xylazine (13 mg kg^−1^, Rompun, Bayer); during and after surgery, animals were kept at 34 °C.

### The STA test

The STA test was conducted in a 30-cm diameter circular Plexiglas arena across at least three sessions (10 min each): (1) habituation to the empty arena; (2) familiarization (24 h later), during which a one-sided sticky tape (four 0.8 × 0.8 cm joint squares of green insulating PVC tape) was placed 3 cm from the wall to maximize a rapid encounter with the tape and to later underline the avoidance behavior; and (3) retrieval to assess avoidance. A second retrieval session was performed in a subset of animals (Figs. 6 and 7). Retention intervals ranged from 24 h to 1 month. Tape location differed between sessions (≥90°) and mice were introduced opposite to the tape. The tape was removed 15–30 s after detachment. Control groups included nonsticky tape exposure (n-STA) and no-tape conditions. Behavior was recorded (videotaped at 30-Hz frame rate, 1,024 × 876 pixel resolution) and analyzed offline.

### Analysis of STA

Behavioral features were extracted from mouse trajectories and activity. The center of mass was extracted using a custom-made MATLAB script (R2020b, Mathworks) based on contrast differentiation; snout position was tracked using DeepLabCut^70^. Trajectories were smoothed with a Savitzky–Golay filter. Behavioral feature extraction combined MATLAB routines and observation.

#### Period from arena entry to tape contact

We measured entrance speed (averaged instantaneous speed during the first 2 s inside the arena) and latency to first passage of the mouse close to the tape (distance tape-body center <8 cm). As PCA indicated an association between higher precontact speed and lower avoidance, we verified that entrance speed and latency-to-contact at retrieval were not correlated (Pearson correlation two-sided test: *r* = 0.032; *P* = 0.735).

Behaviors close to the tape were classified as follows: sniffing (distance tape-snout <4 cm); detour (trajectory deviation <120°, tape-mass center <12 cm); U-turn (approach-retreat sequence with <30° angle, retreat following the same path at ≧15 cm s^−1^); side passage (rearing or walking <8 cm, without directed gaze or sniffing or trajectory change >120°, including stepping over the tape without sticking to it).

During familiarization, mice exhibiting strong avoidance (latency-to-contact >180 s with detours ≧2 or latency-to-contact >100 s with detours ≧3) were excluded (*n* = 18; Extended Data Fig. 1f).

#### Period around the sticking up to the removal of the sticky tape

We quantified parameters related to tape contact and removal: immediate presticking speed (cm s^−1^): averaged instantaneous speed over the 2 s preceding contact; immediate presticking activity labeled as (0: sniffing, 1: retreat postsniffing, 2: walking, 3: rearing, 4: others (for example, grooming or staying still nearby), 5: none); and gaze as (1: directed, 2: last-second gaze (mouse looked upwards while walking and directed its gaze only once very close to the tape), 3: distracted (mouse did not look in the tape direction at contact), 5: none); latency-to-contact (seconds); body part contacting the tape:) 0: snout; 1: forepaw, 2: hindpaw, 3: tail, 5: none); contact duration (total time with the tape glued to the mouse; seconds); time-to-remove (cumulative durations of voluntary movements for tape removal); tape removed passively: 0. Where applicable, indices 0–5 reflected decreasing engagement with the tape; 5 denoted animals that did not tape-stick at retrieval.

#### Period after removal of the sticky-tape

Most mice removed the sticky tape within 10 min (except for 6%). A subset (*n* = 18 of 74) showing avoidance behavior at familiarization were excluded (Extended Data Fig. 1f) as it prevents one-shot learning evaluation.

For complementary parameters and analyses, see Supplementary Information ‘Methods’ and Supplementary Figs. 1–3.

#### Distribution of trajectory in space and time

Mouse trajectories were rotated so the tape aligned at 0° angle. The polar coordinates (center of mass) were binned (radius: 6 bins, 0–15 cm; angle: 17 bins, 0–180°) using absolute angle values to preserve symmetry. Occupation probabilities were normalized by radius to account for the nonuniform surface across two-dimensional bins. Average speed was computed per two-dimensional bin. For each animal, occupation probabilities and speed were calculated from trajectories cumulated up to +30s, +60s and +200 s as long the mouse did not stick to the tape (*n* = 36, *n* = 34, *n* = 20, respectively, with *n*_total_ = 56 mice). In n-STA, all trajectories were included for all timepoints (*n*_total_ = 22).

A Linear mixed model was fitted to the occupation probabilities and speed (restricted maximum likelihood algorithm), with the following factors: Cluster (n-STA versus STA), Zone (three equal areas: near tape, 90°, opposite), Time (three timepoints defined as above). The cluster variable was the mouse ID. We have: Occupation Probability (or Speed) ~1 + Cluster + Timepoints + Zone + Cluster:Timepoints + Cluster:Zone + Timepoints:Zone + Cluster:Timepoints:Zone + (1|Mouse ID).

The proportion of time spent immobile (instantaneous speed <2 cm s^−1^), near the walls (greater than two-thirds of arena radius) and in half of the arena opposite to or containing the tape were estimated at various timepoints.

#### Avoidance index

To establish an avoidance index for each mouse, we quantified parameters measurable at both familiarization and retrieval: (latency-to-contact, detours, sniffings, U-turns, side passages, immediate presticking speed and/or gaze, initial contact body part). Other parameters correlated highly with latency-to-contact (percentage of time near the walls or tape, immobility) and mostly meaningless for short latency-to-contact were excluded (Extended Data Fig. 1b). We clipped the number of sniffings, detours and side passages at 2, and U-turns at 1, to reflect qualitative differences in behaviors and avoid biases associated to heterogeneity in latency-to-contact; these thresholds maximized the separation between familiarization and retrieval behaviors (Extended Data Fig. 1e). PCA was run on *Z*-scored parameters at retrieval (*n* = 118 mice, with retention interval of 24 h to 1 month; Figs. 1 and 2). The first two PCs explaining 72% of the variance (57.7% and 14.3%, respectively), were used to define the avoidance index (Extended Data Fig. 2a–c). These two PCs were conserved throughout the study and used for projecting other behavioral data. When projecting the mouse scores on the first two PCs, two main axes of variation of data dispersion, aligned approximately onto two diagonals (a weighted sum of PCA loadings yielded respectively: 0.35 × PC1 + 0.27 × PC2 and 0.31 × PC1–0.33 × PC2). For simplicity, we kept the diagonal axis {PC1 − PC2} and {PC1 + PC2} and evaluated their respective weights by quantifying the steepness of their evolution between familiarization and retrieval (Extended Data Fig. 2c). The vector {PC1 − PC2} gives weight to the number of detours, sniffings, side passages and U-turns with {PC1 + PC2} giving weight to contact configuration and latency.

The avoidance index was defined as follows:

$${\rm{Avoidance}}\,{\rm{index}}={\rm{score}}\left(\left\{{\rm{PC}}1-{\rm{PC}}2\right\}\right)+w\times {\rm{score}}\left(\left\{{\rm{PC}}1+{\rm{PC}}2\right\}\right),$$

with w = 0.45, the ratio: $\frac{{\mathrm{Score}\{\mathrm{PC}1-\mathrm{PC}2\}}_{{\rm{R}}1}}{{\mathrm{Score}\{\mathrm{PC}1-\mathrm{PC}2\}}_{\mathrm{Fam}}}/\frac{{\mathrm{Score}\{\mathrm{PC}1+\mathrm{PC}2\}}_{{\rm{R}}1}}{{\mathrm{Score}\{\mathrm{PC}1+\mathrm{PC}2\}}_{\mathrm{Fam}}}$

Avoidance index was correlated strongly with PC1, whether the input data was or not truncated (Extended Data Fig. 2e). Mice with avoidance index > 0 were classified as avoiders—a threshold that reliably distinguished avoidance from nonavoidance behavior (Extended Data Fig. 2d).

### New object recognition, rotarod and cross-maze tasks

See Supplementary Information ‘Methods’.

### Enzyme-linked immunosorbent assay for corticosterone quantification

Blood samples were collected in EDTA-coated tubes (Microvette 200 K3E, Sarstedt) 30 min before and 20 min after familiarization and retrieval sessions, during light cycle (1:00–7:00 pm). Blood was sampled from the submandibular vein under isoflurane anesthesia, centrifuged (4 °C, 10 min, 5,000*g*), and the supernatant stored at −80 °C. Corticosterone levels (pg ml^−1^) were measured according to manufacturer’s instructions (Biotechne, Corticosterone Parameter Assay Kit, cat. no. KGE009) using a 1:60 dilution factor, with absorbance read at 450 nm (Spark, Tecan). All assays were performed in duplicate and concentrations were determined from logistic regression–based standard curves.

### Cortical viral injections

Viral injections were performed in S2 cortex of C57BL/6J mice, CB1R^flox/flox^ and Drd2^loxP/loxP^ mice (P35–40 days). To express ChR2 in L5 pyramidal neurons, AAV5-CAMKII-ChR2-mCherry (Addgene cat. no. 26975, titer: 10^13^ particles ml^−1^) was delivered unilaterally. To selectively ablate D2R or CB1R in L5 pyramidal neurons, AAV1.CMV.HI.eGFP-Cre.WPRE.SV40 (Addgene cat. no. 105545, titer: 10^13^ particles ml^−1^, dilution 1:5) was delivered bilaterally in Drd2^loxP/loxP^ or CB1R^flox/flox^ mice. Injection of AAV1.CMV.HI.eGFP. WPRE.SV40 (Addgene cat. no. 105530, titer: 10^13^ particles ml^−1^, dilution 1:5) was used as a control.

Two craniotomies were performed at the coordinates relative to bregma: (−3.85 mm lateral, +0.5 mm anterior, −3.05 mm deep from the skull surface at bregma) and (−4.05 mm lateral, −0.5 mm posterior, −2.95 mm deep). AAV (350 nl per site) was injected using a 5-µl Hamilton syringe at 70–80 nl min^−1^. In vivo behavior and/or electrophysiology, and in vitro patch-clamp were performed 3–5 weeks postinjection.

### In vivo corticostriatal ChR2-evoked LFP recordings and analyses

Corticostriatal ChR2-evoked LFP experiments were performed as described in ref. ^38^. Two weeks after viral injection, an optrode was implanted. The optrode consisted of a stainless-steel double-strand wire (California Fine Wire): each strand, soldered to a four-pin connector, was twisted and glued along a flat-cleaved 5-mm length optic fiber of 0.39 numerical aperture (NA) and 200-μm core diameter (cat. no. CFMLC 12L05, Thorlabs) and aligned with the fiber tip (±200 μm). The optrode was implanted in DLS (−2.9 mm lateral; −0.1 mm posterior; 3.7–3.9 mm deep). Ground and reference screws (Antrin Miniature Specialties) were screwed over the cerebellum. The implant was cemented to the skull using Superbond C&B dental cement (Sun Medical), insulated with self-curing acrylic (Dentalon Plus, Kulzer) and protected by a skull-mounted copper mesh circlet.

Extracellular signals were amplified, multiplexed and acquired at 40 kHz (cat. no. KJE-1001, Amplipex). ChR2 was activated with a 475-nm laser diode (cat. no. FLS-475, DIPSI) using an implanted optic fiber cannula (ceramic ferrule of 1.25 mm diameter and 6.4 mm length; ThorLabs) connected to a FC/PC patch cable with similar ferrule diameter. ChR2-LFPs were recorded 10 min before and at 2 h and 4 h after training in awake mice. Light was delivered for 10 min at 0.14 Hz (square pulses of 300 μs on average, range: 150–800 μs) at 7–15 mW at the fiber tip, controlled by an Arduino UNO.

Data were analyzed in MATLAB. ChR2-LFPs were extracted and conserved when the first component slope was clearly identified, smoothed (150-μs moving mean) and realigned to zero by subtracting the average voltage of the 375 μs prestimulation. Each measurement of ChR2-LFP was averaged from six consecutive responses, and second phase amplitude was normalized to the first. Synaptic changes were assessed per mouse was estimated using repeated-measures analysis of variance (ANOVA) and Tukey post hoc tests, on the 10–15 batch measurements per session.

In vivo pharmacology was performed in cannula-equipped mice (‘Canula implants’), using 1 μM TTX (Tocris) in saline, combined with ChR2-LFP recordings. In vitro, an optic fiber was inserted at the surface of horizontal slices in DLS (‘Ex vivo patch-clamp recordings’). LFPs were recorded at 20 kHz (cat. no. EPC10, HEKA Elektronik) with a 3–5 MΩ artificial cerebrospinal fluid (ACSF)-filled glass pipette, placed within the illuminated area. After a 10-min baseline recording in ACSF, slices were perfused sequentially with D-AP5 (50 μM), D-AP5 + CNQX (20 μM)or D-AP5 + CNQX + TTX (1 μM) (Tocris).

### Fiber photometry

GRAB_eCB2.0_ (AAV2/9-hSyn-eCB2.0, 9.5 × 10^13^ viral genomes ml^−1^; WZ Biosciences) or GRAB_eCB2.0mut_ (pAAV-hSyn-DIO-GRABeCB2.0mut, 2.45 × 10^13^ viral genomes ml^−1^; WZ Biosciences) biosensor^40,41^ was injected in DLS (300 nl at 50 nl min^−1^ and 400 nl at 70 nl min^−1^, respectively). Stereotaxic coordinates were: antero-posterior (AP) –0.3 mm, medio-lateral (ML) ±2.8 mm, dorso-ventral (DV) −4.25 mm. An optical fiber (200 μm, 0.5 NA; Doric Lenses) was implanted above the injection site (AP –0.3 mm, ML ±2.8 mm, DV −2.85 mm) and secured with adhesive cement and dental acrylic.

Fiber photometry was performed with a Doric iFMC system using multiplexed 405 nm (isosbestic) and 465 nm (eCB2.0-dependent) excitation. Emitted photons were detected by a photomultiplier tube and digitized at 1.2 kHz. The signal was low-pass filtered (10 Hz), corrected for photobleaching (double-exponential fit) and motion artifacts (405–465 nm linear regression) and expressed as Δ*F*/F_0_ trace and *Z*-scored. Event-aligned analyses (−10 s to +20 s) were computed relative to the start of contact with the sticky tape, with random timepoints as an internal control. Analyses used custom Matlab scripts (MathWorks).

### Cannulas implants and in vivo microinjections

Double guide cannulas (33GA, Bilaney) were implanted per hemisphere at (+0.55 mm anterior; −2.9 mm lateral) and (−0.4 mm posterior; −3.1 mm lateral) at 2 mm below the skull. Guide cannula dived down to 0.5 mm from the skull surface. Internal cannula (33G, Bilaney; 3.5-mm length; 2-mm projection from the guide) were inserted. After anesthesia, bilateral infusion was performed with 10-μl Hamilton syringes pump-connected. D-AP5 (500 μM, saline) or AM251 (5 μM, 100 μM or 500 μM, DMSO 0.5% to 10%) was injected bilaterally (1 μl per site, 0.1 μl min^−1^) into the DLS. Behavioral testing was performed 45 min after the first injection.

### Neuropixels electrophysiological recordings

#### Headplate implantation and Neuropixels recordings

Acute Neuropixels recordings were performed in mice (P40–45) head-fixed using a steel headplate in a low-weight air-lifted carbon cage (65 g, 290-mm diameter; MHC, Neurotar).

During brief isoflurane anesthesia (1.5% maintenance, 100% O_2_), one craniotomy over left S1 cortex was drilled (−0.1 mm AP; −2.9 mm ML), the dura removed, and the craniotomy covered with Kwik Cast (WPI). A DiI-coated Neuropixel Phase 3b^71^ was inserted (10 μm s^−1^) to 3.8–4 mm using a three-axis micromanipulator (Luigs&Neumann). Signals were acquired with SpikeGLX (HHMI/Janelia Research Campus) and split into a spike-band (30-kHz sampling rate, 500-Hz high-pass filter, gain: 500×) and an LFP-band (2.5 kHz, 1,000 Hz low-pass, 250×) using a PXI-e8381 (National Instruments). The electrode tip was used as reference. After 2–3 weeks of habituation, the recording session took place: first, a 15-min baseline, with the head-fixed mouse left in the empty arena, was performed; the mouse was then subjected to the STA familiarization and recording continued up to 30 min postfamiliarization.

#### Data analysis

Spike sorting was performed offline using Kilosort-2.5 (refs. ^72,73^) and curated manually with phy (https://github.com/kwikteam/phy). Preprocessing included high-pass filtering (300 Hz), common average referencing and whitening. Clusters with ≥50 spikes were inspected and discarded if the template waveform was near noise or nonphysiological. Units were classified based on amplitude, signal-to-noise ratio and refractory period contamination and merges were checked. Double-counted spikes from templates fitting residuals were removed using a 0.16-ms temporal and 50-μm spatial window. Analyses were performed on units with minimal refractory period contamination and nontruncated spike amplitude distribution.

#### Mouse habituation to MHC

The mouse was habituated to the MHC for 2–3 weeks. On the first day, mice were left 5 min inside the cage, with the air pressure on, without head-fixation. Then, mice were head-fixated (with a 20° tilted head-clamp) and left for 15 min. Training duration increased progressively (on the first 3 days, 15 min, 30 min and 45 min; then on subsequent days, alternation between 1 h and 2 h of training), with one session per day. Starting on the fifth session, small objects were placed in the border or center of the cage forming obstacles to improve the anti-collision abilities of mice. After eight to ten sessions, headplate position relative to the MHC head-fixation bar was rotated by 90°. This position was preferred over time as mice better followed the natural circular movement of the cage and anticipated collisions with objects. Mice were also habituated to the presence of the electrophysiological apparatus and light (to mimic the light needed for probe insertion). On the day preceding the Neuropixels recordings, a special floor made of corrugated paperboard was added, which allowed a finer grip.

### Ex vivo whole-cell patch-clamp

#### Brain slice preparations and SPN whole-cell recordings

Horizontal and parasagittal (30°) brain slices were used to stimulate cortical inputs from somatosensory or anterior cingulate cortex, to the DLS or DMS, respectively^31^. Slices (270-µm and 300-µm thick, respectively) were cut using a microtome (cat. no. 7000smz-2, Campden Instruments) in an ice-cold cutting ACSF solution 95% O_2_/5% CO_2_-bubbled containing: 125 mM NaCl, 2.5 KCl mM, 25 mM glucose, 25 mM NaHCO_3_, 1.25 mM NaH_2_PO_4_, 2 mM CaCl_2_, 1 mM MgCl_2_.

Borosilicate glass pipettes (4–7 MΩ) were filled with: 122 mM K-gluconate, 13 mM KCl, 10 mM HEPES, 10 mM phosphocreatine, 4 mM Mg-ATP, 0.3 mM Na-GTP, 0.3 mM EGTA (pH 7.35, KOH). Signals were recorded using EPC10-2 amplifiers (HEKA Elektronik) and sampled at 10 kHz (Patchmaster v2x32, HEKA Elektronik). All recordings were performed at 34 °C (Bath-controller V, Luigs&Neumann).

#### STDP: protocols and offline analysis

Electrical stimulations were delivered with concentric bipolar electrodes (Phymep) placed in cortical layer 5 using a monophasic constant-current stimulator (ISO-Flex, AMPI). Currents were set to evoke 50–300pA EPSCs at 0.1 Hz in SPNs held at −70 mV during the STDP protocol. STDP protocols consisted of 15 or 100 post–pre pairings (Δ*t*_STDP_ = −15ms) at 1 Hz or 2.5 Hz^19–22^. Postsynaptic stimulation (depolarizing current step, 30 ms) elicited 1 and 2 spikes for 100 and 15 pairings, respectively. Recordings included a 10-min baseline and ≥30-min postprotocol. Input and series resistances were monitored and cells were excluded if resistances varied by >20%. Offline analysis was performed with custom MATLAB (MathWorks) code.

#### Ex vivo experiments measuring occlusion or saturation

Mice undergoing no-tape or STA tests were assigned to postfamiliarization or postretrieval groups. Ex vivo whole-cell recordings were performed 30 min (or 24 h) after task completion. eCB-LTP and NMDAR-LTP occlusion or saturation was tested with 15 or 100 post–pre pairings (1 Hz, Δ*t*_STDP_ = −15ms). In most cases, eCB-LTP and NMDAR-LTP ex vivo expressions were tested from the same animals; ‘*n*’ refers to a single-cell or slice.

### Histology

Injection sites and viral expression were verified post hoc. Brains were postfixed in 4% paraformaldehyde, cryoprotected in 30% sucrose, and cut coronally (80 µm) in 0.1 M potassium–PBS (pH 7.4). Slices were stained with 4′,6-diamidino-2-phenylindole, mounted with Fluoromount (Thermofisher), imaged using Axiozoom (Zeiss) and processed in ImageJ software (https://imagej.net/ij/).

### Theoretical tools for plasticity induction estimation

#### Mathematical model

Synaptic plasticity expression was estimated using a mathematical model of the effect of input spikes on the concentration of the main signaling actors involved in corticostriatal STDP^19–21^. The biophysical model describes molecular pathways and ionic dynamics through nonlinear ordinary differential equations comprising, for example, glutamate receptors, VSCC, CB1R or IP3R, enzymes including FAAH, CaMKII kinase or DAG lipase, and relevant metabolites, molecules or ions including glutamate, Ca^2+^, DAG, 2-AG or IP_3_. Postsynaptic CaMKII phosphorylation serves as a proxy for NMDAR-dependent STDP-LTP, whereas presynaptic CB1R activation by endocannabinoids (2-AG and anandamide) indexes endocannabinoid-dependent plasticity, with intermediate CB1R activation triggering eCB-LTD and high CB1R activation inducing eCB-LTP^19^.

The model was calibrated and validated in ex vivo STDP experiments^14,19,21^ and extended across different experimental contexts^20,39^. We used the original version of the model^21^ without modifying equations or parameters, except for glutamate release per presynaptic spike Glu_max_ and the duration of the postsynaptic depolarization associated to postsynaptic spike DP_dur_, to reflect in vivo versus ex vivo activity levels. We decreased Glu_max_ to 1.0 mM as ex vivo presynaptic stimulations induce a large amount of synchronized presynaptic inputs whereas in vivo recording gives access to single-cell spike trains. Postsynaptic depolarization duration was reduced from 30 ms (ex vivo) to 14 ms.

#### Numerical simulation

The location of each unit was inferred from its electrode position along the Neuropixels 1.0 probe, enabling classification as cortical or striatal. Corticostriatal neuron pairs were evaluated for plasticity expression, using recorded spike trains as inputs to the model. Simulations were performed in Python 3.10.12 using Runge-Kutta 4-5 solver from (scipy package)^74^, and optimized with numba^75^, with data management using Hydronaut (https://gitlab.inria.fr/jrye/hydronaut). Plasticity expression was evaluated 5 min before to 5 min after contact time.

#### The plasticitymeter

The presynaptic weight ${w}_{\mathrm{pre}}$ is estimated from the calcium amount and CB1R bound to endocannabinoids, according to the differential equation $d{w}_{\mathrm{pre}}/{dt}=(\Omega -{w}_{{pre}})/\sigma$, where the timescale σ decreases with calcium concentration (and defines the delay for ex vivo plasticity expression) and Ω is a CB1R-dependent function defining ${w}_{\mathrm{pre}}$ variation from synaptic inputs. Ω is negative for intermediate 2-AG level binding to CB1R, leading to LTD, and Ω becomes positive when the 2-AG binding level exceeds a threshold, leading to LTP^21^.

We combined the values of Ω and σ into a quantifier of plasticity expression termed ‘plasticitymeter.’ Spike trains were binned into *N* consecutive nonoverlapping 0.2-s bins. In each 0.2-s bin, we defined a binary variable ${\pi }_{i,j}\in \{\mathrm{0,1}\}$ for each neuronal pair (i,j), set to 1 if at least one plasticity event occurred, and 0 otherwise. A plasticity event (LTP or LTD) arises when Ω is nonzero and σ is low enough to maintain a synaptic weight change (here, σ<100). We kept separate counts of eCB-LTP (Ω>0) and eCB-LTD (Ω<0) events.

We defined the plasticitymeter P of a subpopulation A of pairs as the mean plasticitymeter on A, renormalized by the mean plasticitymeter over all pairs. The plasticitymeter P of subpopulation A at bin k is given by

$${P}^{k}=\sum _{\left(i,j\right)\in A}\frac{{\pi }_{i,j}^{k}}{\mathop{\sum }\limits_{m=1}^{N}{\pi }_{i,j}^{k}+1}$$

(the numerator was slightly altered by adding a control term ‘+1’ to avoid divisions by 0). We used the temporal mean $\mu =1/N\sum _{k}{P}^{k}$ and SD s of plasticitymeter values to obtain its *z* score ${z}_{k}=({P}^{k}-\mu )/s$.

#### Subselection of neuron pairs

We restricted analyses to pairs more likely to be connected, given the large number of corticostriatal combinations in Neuropixels data. Pairwise correlations $\left(i,j\right)$ were quantified during sticky tape contact:

$${C}_{i,j}={p}_{i,j}^{c}/\left({n}_{i}^{c}+{n}_{j}^{c}\right)$$

where

- ${n}_{i}^{c}$: number of spikes in the train of neuron *i*, during the first 20 s of contact
- ${p}_{i,j}^{c}$: number of pre–post or post–pre spike pairs in intervals ≤0.2 s, during the contact or baseline epochs.

The neuron pairs (i,j) with a ${C}_{i,j}$ within the top 5% formed the active pairs (Extended Data Fig. 6c).

### Statistics and reproducibility

Results are expressed as mean ± s.e.m. All boxplots show the median as the central mark; the box spans from the 25th to the 75th percentile (interquartile range (IQR)), and whiskers extend to 1.5× IQR. The results of every statistical test, linked to the corresponding figure and panel, are reported in ‘Statistics’ in Supplementary Information ‘Methods’. Statistical significance was assessed using MATLAB (MathWorks) or Jamovi (http://www.jamovi.org). All two-sample and one-sample tests were two-tailed. No statistical method was used to predetermine sample size. Statistical source data and code to reproduce statistical tests and plots are available in the GitHub repository: https://github.com/cpiette95/one_shot_learning.git.

Experiments were not randomized. Experiments and analyses were performed blinded in all transgenic mice, and for ex vivo patch-clamp. Experimenters were blinded to outcome assesment.

No data were excluded from the analyses, except mice exhibiting strong avoidance at familiarization (*n* = 18; Extended Data Fig. 1f) and ex vivo SPNs displaying input and series resistances varying by >20%.

### Reporting summary

Further information on research design is available in the Nature Portfolio Reporting Summary linked to this article.

## Online content

Any methods, additional references, Nature Portfolio reporting summaries, source data, extended data, supplementary information, acknowledgements, peer review information; details of author contributions and competing interests; and statements of data and code availability are available at https://doi.org/10.1038/s41593-026-02392-z.

## Supplementary information

Supplementary Information Supplementary Methods (and Statistics), Note and Figs. 1–3.

Reporting Summary

## Data Availability

All data required to reproduce all statistical tests and plots in the paper are available at via GitHub at https://github.com/cpiette95/one_shot_learning.git. Any further materials will be made available upon reasonable request.

## Code availability

All code required to reproduce all statistical tests and plots in the paper are available at via GitHub at https://github.com/cpiette95/one_shot_learning.git. Any further materials will be made available upon reasonable request.
