# Supplementary material for: Striatal endocannabinoids drive one-shot learning
Source: Nat Neurosci. 2026 Aug 7;29(10):2523–37. doi: 10.1038/s41593-026-02392-z (PMC13633119; doi:10.1038/s41593-026-02392-z)
Supplement: Supplementary file 1 [file 41593_2026_2392_MOESM1_ESM.pdf]

nature neuroscience

Supplementary information

https://doi.org/10.1038/s41593-026-02392-z

Striatal endocannabinoids drive one-shot learning

In the format provided by the authors and unedited

Supplementary information

Supplementary Methods

Behavioral tests used in the 1-month interval between familiarization and retrieval (related to the **Figure 2)**

Object recognition memory: novel object recognition (NOR) task.

The NOR task with four objects (Lego blocks) was run in a square Plexiglas arena (40x40cm) and consisted of three consecutive sessions at 24 hours intervals: (1) 10 min habituation to the empty arena, (2) 3 min familiarization to objects, and (3) 5 min retrieval session in which a novel object replaced one of the four objects. The object discrimination ratio was calculated as the ratio of the time spent exploring the novel object at retrieval over either (a) the average time spent on the four objects at familiarization or (b) the average time spent on the three familiar objects at retrieval.

Procedural learning: accelerating rotarod.

Mice were trained on the accelerating rotarod (model 8200, Panlab) in 5 consecutive daily sessions of 5 trials (acceleration from 4 to 40rpm during 300 seconds). Between each trial, mice were placed in their homecage to rest for 15 minutes. Performance was assessed by subtracting the average latency of the last two trials of Day 1 (early state) or of Day 5 (late stage) to the average latency of the first two trials of Day 1.

Reward-guided learning: cross-maze task.

Mice access *ad libitum* to water containing 4% citric acid over one week. The north arm was used as starting point during the habituation/training period while entry to the south arm was blocked. During a 20 minutes habituation, the preferred west or east arm was determined by counting the number of visits. Then, the mouse was left another 20 minutes in the cross-maze with a cup of water at the extremity of the non-preferred arm (the baited arm). The training started on the next day and consisted of three consecutive daily sessions of 10 trials each, and the proportion of visits to the baited arm was quantified. The reward consisted of a drop of water (20µL). On the last day, a single probe test, in which the entry point of the mouse into the maze was shifted to the south arm (north arm was blocked) allowed to determine the response or place strategy of the mouse.

Generalization of the eCB-LTP involvement in the early stages of the accelerating rotarod (related to **Extended Data Figure 8**).

Ex vivo patch-clamp occlusion experiments

Mice were trained on the rotarod (model 47800, Ugo Basile, Gemonio, Italy), either in the non-accelerating mode (max. 300 seconds, at 4 rpm, single-session) or in the accelerating mode (from 4 to 40 rpm during 300 seconds maximum) in 2 daily sessions (separated by 15 min). The latency to fall from the rod or turn passively for two consecutive cycles (considered the end of the trial) is reported.

Training of conditional knock-out mice

Mice were trained on the accelerating rotarod (model 8200, Panlab, Barcelona, Spain) with 2 daily sessions during 10 days (from 10 to 60 rpm during 300 seconds maximum), separated by a 15-min resting period. The latency to fall from the rod or turn passively for two consecutive cycles (considered the end of the trial) is reported for each animal.

Statistics

For **Fig. 1:** (**b-e**), Comparison of behavioral features between STA (grey, n=56 mice) and n-STA (blue, n=22 mice) for familiarization and retrieval. **b**, Contingency Tables: between retrieval ≥1 detour: χ^2=21.1, *p*<0.001 and ≥2 detours: χ^2=18.1, *p*<0.001; between familiarization ≥1 detour: χ^2=0.414, *p*=0.520 and ≥2 detours: χ^2=0.084 *p*=0.771. Two-sided McNemar-Test for paired samples: within n-STA for ≥1 detour: χ^2=0.143; *p*=0.705 and ≥2 detours: χ^2=18, *p*<0.001; within STA for ≥1 detour: χ^2=23.1, *p*<0.001 and ≥2 detours: χ^2=11.6, *p*<0.001. **c**, Repeated-measures ANOVA with two-sided t-tests with Tukey correction. Between-Subjects (nSTA *vs.* STA): *F*=16.2, *p*<0.001; familiarization-retrieval: *F*=17, *p*<0.001; Combined: *F*=16.3, *p*<0.001. Post-hoc t-tests with Tukey correction: STA familiarization/retrieval: *t*=-7.7, *p*<0.001; nSTA familiarization/retrieval: *t*=-0.0482, *p*=1.0; familiarization nSTA/STA: *t*=0.0209, *p*=1.0; retrieval nSTA/STA: *t*=4.069, *p*<0.001. **d**, Repeated-measures ANOVA with two-sided t-tests with Tukey correction. Between-Subjects (nSTA *vs.* STA): *F*=13.1, *p*<0.001; familiarization-retrieval: *F*=16, *p*<0.001; Combined: *F*=13.4 *p*<0.001. Post-hoc t-tests with Tukey correction: STA familiarization/retrieval: *t*=-7.2, *p*<0.001; nSTA familiarization/retrieval: *t*=-0.197, *p*=0.997; familiarization nSTA/STA: *t*=0.281, *p*=0.992; retrieval nSTA/STA: *t*=3.673, *p*=0.002. **e**, Log-rank test (Mantel-Haenszel Hazard ratio, MHZ): within n-STA: MHZ=1.14; *p*=0.79; within STA: MHZ=5.74; *p*<0.001; between familiarization: MHZ=1.28; *p*=0.47; between retrieval: MHZ=8.36; *p*<0.001. **f**, Paired two-sample t-test: *t*=3.23; *p*=0.002. **i**, Repeated-measures ANOVA with two-sided t-tests with Tukey correction on avoidance index. Between-Subjects (nSTA *vs.* STA): *F*=29.2, *p*<0.001; familiarization-retrieval: *F*=16.2, *p*<0.001; Combined: *F*=13.0 *p*<0.001. Post-hoc t-tests with Tukey correction: STA familiarization/retrieval: *t*=-7.18, *p*<0.001; nSTA familiarization/retrieval: *t*=-0.245, *p*=0.995; familiarization nSTA/STA: *t*=1.856, *p*=0.256; retrieval nSTA/STA: *t*=5.08, *p*<0.001. Two-sided McNemar test for avoider rate: within STA: χ^2=59.4, *p*<0.001; within nSTA: χ^2=0 *p*=1. **j**, Two-sided χ^2-test on avoider rate: χ^2=6.14, *p*=0.105 for contact duration (right); χ^2=4.71, *p*=0.194 for time-to-remove (left).

For **Fig. 2: b**, Paired t-tests corrected with Benjamini-Hochberg correction. Latency-to-contact: 24h: *p*<0.001, 72h: *p*=0.01, 1 week: *p*=0.01, 1 month: *p*=0.01 and 1month+extra-tasks: *p*=0.0982. Number of detours: 24h: *p*<0.001, 72h: *p*=0.0086, 1 week: *p*=0.0163, 1 month: *p*=0.0163 and 1month+extra-tasks: *p*=0.0187. Two-sample t-test at 1 month with or without extra-task learning (latency-to-contact: *t*=0.364, *p*=0.718; detours: *t*=0.142, *p*=0.888). **c**, Paired t-tests with Benjamini-Hochberg correction. Avoidance index: 24h: *p*<0.001, 72h: *p*<0.001, 1 week: *p*=0.00624, 1 month: *p*=0.00121 and 1month+ extra-tasks: *p*=0.00624. Two-sample t-test between 1 month with/without extra-task: *t*=0.366, *p*=0.717. McNemar test for percentage of avoiders: 24h: *p*<0.001, 72h: *p*=0.0198, 1 week: *p*=0.0198, 1 month: *p*=0.0111 and 1month+ extra-tasks:

p=0.0233. **d**, ANCOVA on avoidance index with two categorical variables {retention interval condition and contact duration (≤25 seconds)}: no effect of each variable (*F*=0.910, *p*=0.461 and *F*=1.664, *p*=0.200 respectively), or when combined (*F*=0.876, *p*=0.481).

For **Fig. 3**: **b**, One-Way ANOVA: *F*=2.37, *p*=0.130. One-sample t-test for LTP significance: no tape: *t*=0.286, *p*=0.781; n-STA: *t*=0.446, *p*=0.671; STA: *t*=2.28, *p*=0.0327. **c**, Boxplot representation, with center line, median; box limits, upper and lower quartiles; whiskers, 1.5x interquartile range; points, outliers. Two-sample t-test: *t*=-2.33; *p*=0.0295. **d**, Linear Mixed Model with Tukey correction applied to post hoc two-sided t-tests; Linear Mixed Model with fixed effect of {Condition: *F*=7.43, *p*<0.001; Session: *F*=1.65; *p*=0.165; Condition*Session: *F*=4.41, *p*<0.001). Fixed Effect of Parameter Estimates {long *vs.* short contacts: t=-4.0110, p<0.001; no-tape *vs.* short: t=-3.826, p<0.001; nSTA *vs.* short: t=-2.961, p=0.005; Fam+2h-Fam: t=0.0151, p=0.988; Fam+24h-Fam: t=1.538, p=0.127; R1+2h-Fam: *t*=1.505, *p*=0.135; R1+24h-Fam: t=1.914, p=0.058; long *vs.* short*(Fam+2h-Fam): *t*=-1.3169; *p*=0.190; long *vs.* short*(Fam+24h-Fam): t=-2.827, p=0.005; long vs. short*(R1+2h-Fam): t=-2.533, p=0.012; long *vs.* short*(R1+24h-Fam): t=-4.710, p<0.001. no-tape *vs.* short*(Fam+2h-Fam): *t*=-0.602; *p*=0.549; no-tape *vs.* short*(Fam+24h-Fam): t=-2.340, p=0.018; no-tape *vs.* short*(R1+2h-Fam): t=-4.112, p<0.001; no-tape *vs.* short*(R1+24h-Fam): t=-4.000, p<0.001. nSTA *vs.* short*(Fam+2h-Fam): *t*=-0.200; *p*=0.842; nSTA *vs.* short*(Fam+24h-Fam): t=-1.821, p=0.071; nSTA *vs.* short*(R1+2h-Fam): t=-2.267, p=0.025; nSTA *vs.* short*(R1+24h-Fam): t=-4.775, p<0.001. **e**, Two-sided one-sample Student t-test and ANCOVA on avoidance with two control binary variables {significant LTP and contact duration (≤20 seconds)}: no effect of each control variable (*F*=0.006 and *F*=0.586 respectively), effect of combined control variables (*F*=4.83, *p*=0.041).

For **Fig. 4: b**, Two-sided two-sample Mann-Whitney U-test between the areas under curve for short (n=4 recordings from n=4 mice) *vs.* long contacts (n=5) calculated for the distributions of the proportion of neurons with a most x spikes during contact (*U*=0, *p*=0.0159 for both [0,30] and [0,300] spikes window). Same applied to the proportion of cells with at most x pairings (*U*=0, *p*=0.0159 for both [0,30] and [0,300] pairings window). **f,** One-sided Mann-Whitney U-test: *U*=20.0, *p*=0.0079. **g**, Z-score panel: two-sided two-sample t-test with Benjamini-Hochberg correction ($GRAB_{eCB2.0}$ and $GRAB_{eCB2.0mut}$, n=9 and 6 mice, respectively): bin 6-8 sec: *p*=0.034, 8-10: *p*=0.017, 10-12: *p*=0.022 and 12-14: *p*=0.08. Two-sided paired t-test: t=-3.275, *p*=0.011 (randomly time-shifted traces with $GRAB_{eCB2.0}$) and two-sided Mann-Whitney U-test *p*=0.040 (with $GRAB_{eCB2.0mut}$).

For **Fig. 5**: (**b-d)**: Two-sided one-sample and two-samples independent Student t-tests. **b** and **c**, STDP at 1 Hz in DLS (*t*=3.127, *p*=0.012, n=9 SPNs), in DMS (*t*=-0.1397, *p*=0.892, n=9 SPNs) and 2.5 Hz in DMS (*t*=-0.408, *p*=0.694, n=9 SPNs). **d**, no-tape condition: eCB-LTP (*t*=4.7678, *p*=0.0005, n=13 SPNs at post-familiarization and *t*=5.9277, *p*=0.0001, n=11 SPNs at post-retrieval) and NMDAR-LTP (*t*=3.0762, *p*=0.0276, n=6 SPNs at post-familiarization). STA condition: eCB-LTP at post-familiarization (short contacts, with contact duration ≤ 25

seconds: +30 min, t=0.9074, p=0.3906, n=9 SPNs and +24h, t=-1.1113, p=0.3090, n=7 SPNs; long contacts: t=2.8498, p=0.0191, n=10 SPNs; short *vs.* long contacts: p=0.0445), at post-retrieval ("learnt": t=-1.2466, p=0.2409, n=11 SPNs, "not learnt: t=2.7629, p=0.0327, n=13 SPNs; "learnt" *vs.* "not learnt": p=0.0143; "passive": t=2.6844, p=0.0436, n=6 SPNs). NMDAR-LTP at post-familiarization (short contacts: t=2.2934, p=0.0391, n=14 SPNs; long contacts: t=-1.4896, p=0.1558, n=17 SPNs; short *vs.* long contacts: p=0.078) and at post-retrieval ("learnt": t=7.4260, p=0.00015, n=8 SPNs). **f**, Pearson correlation coefficient (two-sided test). eCB-LTP occlusion: r=0.491, p=0.011 and NMDAR-LTP occlusion: r=-0.300, p=0.035 as a function of contact duration at Fam. **g**, Linear regression model (R-square = 0.239) of eCB-LTP plasticity ratio, as a function of contact duration (t=1.71, p=0.099) and avoidance index (t=-2.80, p=0.009).

For **Fig. 6**: **b**,**g**, Two-sided two-sample Wilcoxon rank test between (**b**) CB1R-Cre (n=10) and CB1R-ctrl (n=9): W=118, p=0.022; (**h**) Drd2-Cre (n=8) and Drd2-ctrl (n=9): W=114 ; p=0.0012. Two-sided one sample Wilcoxon test (against 100%) for CB1R-Cre: W=39; p=0.2754; CB1R-ctrl: W=45; p=0.0039; Drd2-Cre: W=5, p=0.1562; Drd2-ctrl: W=44, p=0.0078. **d,j**, Two-sided two-sample Student t-test (for short contact duration, ≤25 seconds) for latency-to-contact and detours, respectively. $CB1R^{flox/flox}$ (**d**): CB1 short (n=11 Cre and n=16 ctrl): t=3.42, p=0.0215; t=2.98, p=0.006; CB1 long (n=9 Cre and n=13 ctrl): t=0.747, p=0.464; t=1.174, p=0.254. $Drd2^{LoxP/LoxP}$ (**j**): D2 short (n=14 Cre and n=17 ctrl): t=2.86, p=0.0078; t=2.28, p=0.030; D2 long (n=21 Cre and n=14 ctrl): t=-0.670, p=0.508; t=-0.014, p=0.989. **d,j**, two-sided χ^2-test on proportion of mice showing at least 2 detours at retrieval-1. $CB1R^{flox/flox}$ (**d**): CB1 short: χ^2=7.70, p=0.0055; CB1 long: χ^2=2.76, p=0.0964. $Drd2^{LoxP/LoxP}$ (**j**): D2 short: χ^2=6.42, p=0.011; D2 long: χ^2=0.486, p=0.486. **e**,**k,** Two-sided two-sample Student t-tests for avoidance index. $CB1R^{flox/flox}$ (**e**): CB1 short: t=2.82, p=0.0094; CB1 long: t=1.41, p=0.174. $Drd2^{LoxP/LoxP}$ (**k**): D2 short: t=3.01; p=0.005; D2 long: t=-0.684; p=0.498. **e**,**k,** Two-sided χ^2-test on percentage of avoiders. $CB1R^{flox/flox}$ (**e**): CB1 short: χ^2=5.19, p=0.022; CB1 long: χ^2=1.66, p=0.198. $Drd2^{LoxP/LoxP}$ (**k**): D2 short: χ^2=8.01, p=0.004; D2 long: χ^2=1.94, p=0.163. **f**,**l,** Two-sided Linear Mixed Model for short and long contacts. $CB1R^{flox/flox}$ (**f**). *Short contact:* Fixed Effect of {Group: F=7.29, p=0.012; Time: F=13.29, p<0.001; Group*Time: F=2.36, p=0.104}. Fixed Effect of Parameter Estimates {Group: t=-2.70, p=0.012; retrieval-1-familiarization: t=2.47, p=0.017; retrieval-2-familiarization: t=5.15, p<0.001; Group*(retrieval-1-familiarization): t=-2.168, p=0.035; Group*(retrieval-2-familiarization): t=-0.932; p=0.356}. *Long contact*: Fixed Effect of {Group: F=2.23, p=0.151; Time: F=9.50, p<0.001; Group*Time: F=1.9, p=0.316}. Fixed Effect of Parameter Estimates {Group: t=-1.49, p=0.151; retrieval-1-familiarization: t=3.27, p=0.002; retrieval-2-familiarization: t=4.13, p<0.001; Group*(retrieval-1-familiarization): t=-1.34, p=0.188; Group*(retrieval-2-familiarization): t=-1.33; p=0.191}. $Drd2^{LoxP/LoxP}$ (**l**). *Short contact*: Fixed Effect of {Group: F=11.09, p=0.002; Time: F=28.91, p<0.001; Group*Time: F=3.59, p=0.034}. Fixed Effect of Parameter Estimates {Group: t=-3.33, p=0.002; retrieval-1-familiarization: t=4.36, p<0.001; retrieval-2-familiarization: t=7.57, p<0.001; Group*(retrieval-1-familiarization): t=-2.64, p=0.011; Group*(retrieval-1-familiarization):

t=-1.70; p=0.095}. *Long contact*: Fixed Effect of {Group: F=0.228, p=0.636; Time: F=34.7 p<0.001; Group*Time: F=2.22, p=0.117}. Fixed Effect of Parameter Estimates {Group: t=0.477, p=0.636; retrieval-1-familiarization: t=5.78, p<0.001; retrieval-2-familiarization: t=8.07, p<0.001; Group*(retrieval-1-familiarization): t=1.66, p=0.102; Group*(retrieval-2-familiarization): t=1.95, p=0.056}. **m**, Fisher's exact two-sided test on the proportion of LTP (*vs.* no-LTP) for short contacts: p=0.009 and on the proportion of LTD (*vs.* no-LTD) for long contacts: p=0.592.

For **Fig. 7: b**, Two-sided two-sample t-test for latency-to-contact and detours. For short contact (n=6 AM251 and n=8 DMSO): t=2.78, p=0.0166 and t=3.32, p=0.00601 respectively; for long contact (n=15 AM251 and n=12 DMSO): t=0.147, p=0.885 and t=0.814, p=0.424.
Two-sided χ^2-test on proportion of mice showing at least two detours: χ^2=7.024, p=0.00804; χ^2=0.363, p=0.547 for short and long contact, respectively.
Two-sided two-sample t-test for avoidance index: t=2.56, p=0.0250; t=0.550, p=0.587 for short and long contact, respectively. Two-sided χ^2-test on avoider rate: χ^2=7.024, p=0.00804; χ^2=0.270, p=0.6033 for short and long contact, respectively.
Two-sided Linear Mixed Model for short contact. Fixed Effect of {Group: F=4.16, p=0.064; Time: F=12.87, p<0.001; Group*Time: F=1.95, p=0.164}. Fixed Effect of Parameter Estimates {Group: t=-2.039, p=0.064; retrieval-1-familiarization: t=3.907, p<0.001; retrieval-2-familiarization: t=4.757, p<0.001; Group*(retrieval-1-familiarization): t=-1.882; p=0.072; Group*(retrieval-2-familiarization): t=-0.420; p=0.678}.
Two-sided Linear Mixed Model for long contact. Fixed Effect of {Group: F=3.018, p=0.095; Time: F=17.80, p<0.001; Group*Time: F=0.863, p=0.428}. Fixed Effect of Parameter Estimates {Group: t=-1.737, p=0.095; retrieval-1-familiarization: t=4.014, p<0.001; retrieval-2-familiarization: t=5.808, p<0.001; Group*(retrieval-1-familiarization): t=-0.222; p=0.825; Group*(retrieval-2-familiarization): t=-1.240; p=0.221}.
c, Two-sided two-sample t-test for latency-to-contact and detours. For short contact (n=7 D-AP5 and n=5 saline): t=0.297, p=0.773 and t=1.283, p=0.229 respectively; for long contact (n=12 D-AP5 and n=3 saline): t=-0.866, p=0.402 and t=-0.397, p=0.698.
Two-sided χ^2-test on proportion of mice showing at least two detours: χ^2=0.685, p=0.407; χ^2=1.30, p=0.255 for short and long contact, respectively.
Two-sided two-sample t-test for avoidance index: t=1.11, p=0.292; t=-1.29, p=0.219 for short and long contact, respectively. two-sided χ^2-test on avoider rate: χ^2=1.66, p=0.198; χ^2=0.417, p=0.519 for short and long contact, respectively.
Two-sided Linear Mixed Model for short contact. Fixed Effect of {Group: F=0.657, p=0.437; Time: F=20.387, p<0.001; Group*Time: F=0.466, p=0.634}. Fixed Effect of Parameter Estimates {Group: t=-0.810, p=0.437; retrieval-1-familiarization: t=4.667, p<0.001; retrieval-2-familiarization: t=6.108, p<0.001; Group*(retrieval-1-familiarization): t=-0.662; p=0.516; Group*(retrieval-2-familiarization): t=0.278; p=0.784}.
Two-sided Linear Mixed Model for long contact. Fixed Effect of {Group: F=0.239, p=0.633; Time: F=30.20, p<0.001; Group*Time: F=2.433, p=0.108}. Fixed Effect of Parameter Estimates {Group: t=0.489, p=0.633; retrieval-1-familiarization: t=4.478, p<0.001; retrieval-

2-familiarization: $t=7.740$, $p<0.001$; Group*(retrieval-1-familiarization): $t=1.172$; $p=0.252$; Group*(retrieval-2-familiarization): $t=-1.033$; $p=0.311$}.

Supplementary Note

Evaluation of the impact of sticking features at familiarization on retrieval performance

We aimed to detect if the behavior of the mouse during the first encounter with the sticky tape, at familiarization, could affect its behavior during retrieval. To do so, we first extracted parameters describing the sticking modalities and behaviors at familiarization, before, during and after contact with the sticky tape. We then performed multifactorial analyses and found that one-shot learning of STA could emerge regardless of the marked heterogeneity of behaviors recorded at familiarization. We investigated a set of behavioral parameters larger than those briefly described in *Methods*; These are:

Period during the contact with the sticky tape

- the immediate post-sticking activity: stepping back {0: no stepping back, 1: stepping back}, average speed in the 5 seconds after contact (cm/s) and surprise level (scored from 0 to 3, based on mouse reaction, including stepping back, level of interruption in activity, unusual movements).
- latency before first active removal movement (seconds)
- number and percentage of epochs of voluntary movements aiming at tape removal; we classified them as short (<0.5 second), medium (0.5-1 second), long (1-5 seconds), or very long (>5 seconds) active attempts.
- time-to-remove (%): time-to-remove/contact duration
- taped to wall or ground: during contact, the tape could stick both to the mouse and to the ground or walls of the arena {0: <1 seconds, 1: ≥1 seconds}. The total duration spent taped to wall or ground was also considered.
- distance travelled (m)
- time spent immobile (%)
- average and maximal instantaneous speed (cm/s)
- rearings (#)
- time spent grooming (seconds)
- rearings (#) and time spent grooming (seconds) in the first minute following sticking, since 6% of mice did not successfully remove the tape.

Period after removal of the sticky-tape

- maximal speed in the ten seconds following sticky tape removal (cm/s)
- number of sniffings and detours (#)
- number of passages on the tape in the case when this one becomes stuck to the ground as a result of removal.

During the first minute following sticky tape removal (a preliminary comparison was made considering the first three minutes), we quantified:

- time spent immobile (%)
- time spent near the walls (%)
- time spent in the disk centered on the last position of the sticky tape and radius 8 cm (%)
- rearings (#)
- time spent grooming (seconds)

We first considered possible correlations between individual parameters evaluated at familiarization and the latency-to-contact, number of detours and avoidance index at retrieval (**Supplementary Figure 1**). We also performed independent t-tests using for binary classification, a avoidance index ≥0 or number of detours ≥2. We considered all mice or only those with short contact duration (≤30 seconds). We found that the time spent grooming (either 1 min from contact onset or 1 min post-removal) was significantly elevated at familiarization in mice that would subsequently show a clear avoidance response. In the short contact duration cases, we also noted that the percentage of mice that did not step back immediately after sticking to the tape was elevated in mice that showed less than two detours at retrieval (64 %, n=31 *vs*. 39%, n=48, Chi-squared test: χ^2=4.68, *p*=0.030). In the same vein, lower surprise levels were found on average in mice showing less than two detours at retrieval (Chi-squared test: χ^2=10.8, *p*=0.013).

To capture possible dependences between parameters and extract a contact configuration that would favor one-shot learning, we performed a multi-dimensional analysis (**Supplementary Figure 2** and **3**). We considered 19 parameters estimated at familiarization and valid for all mice: {1. latency-to-contact (s), 2. time immobile in other half before contact (%), 3. detours (#), 4. activity pre-contact, 5. first contact location (body part), 6. longest contact location, 7. tapped to the wall/ground (0/1), 8. stepping-back (0/1), 9. contact duration (seconds), 10. maximal speed during contact (cm/s), 11. time-to-remove (seconds), 12. time-to-remove (%), 13. active attempts >5 seconds to remove the tape (#), 14. attempts < 0.5 second (%), 15. attempts [0.5-5 seconds] (%), 16. grooming 1min after contact onset (seconds), 17. rearings 1 minute after contact onset (#), 18. detours post-removal (#), 19. non-sticky tape contacts post-removal (#)}. We then performed a factor analysis on mixed data (FAMD), using R-packages (*FactoMineR and factoextra*). Projections on the first two FAMD principal components of each individual parameter (**Supplementary Figure 2)**. We did not identify any clear hotspot from the mapping of the avoidance index onto the first FAMD principal components. A linear regression analysis was performed to extract dimensions that could predict the avoidance index at retrieval. When considering all mice on the first five dimensions (which explained 50.3% of the variance), it yielded a poor fit (r=0.252). A significant coefficient was found for the fifth principal component (*t*=-2.262, *p*=0.026). Grooming time 1 min after contact onset (contribution =15.39) and number of passages on the non-sticky tape (contribution =23.70) contributed the most to the fifth principal component.

We performed the same multifactorial analysis specifically on mice with short contact duration (≤30 seconds) (**Supplementary Figure 3**). We used 23 parameters (17/23 similar to the previous analysis): {1. latency-to-contact (seconds), 2. time immobile in the other half before contact (%), 3. detours (#), 4. activity pre-contact, 5. first contact location (body part), 6. longest contact location, 7. tapped to the wall/ground (0/1), 8. stepping-back (0/1), 9. contact duration (s), 10. maximal speed during contact (cm/s), 11. time-to-remove (seconds), 12. time-to-remove (%), 13. active attempts >5 seconds to remove the tape (#), 14. attempts <0.5 seconds (%), 15. attempts [0.5-5 seconds] (%), 16. time spent immobile during contact (%), 17. maximal speed in the first 10 seconds post-removal (cm/s), 18. time spent immobile 1 min post-removal

(%), 19. time spent in scotch zone 1 min post-removal (%), 20. rearings 1 min post-removal (#), 21. grooming 1 min post-removal (seconds), 22. detours post-removal (#), 23. non-sticky tape contact post-removal (#)}. The linear regression performed on the first five dimensions (explaining 50.2% of the variance) poorly predicted the avoidance index at retrieval (r=0.266), with no significant regression coefficients.

We tested non-linear classification algorithms either directly on 23 parameters of familiarization or on the first five FAMD components. The classification boundary was set according to the avoider rate boundary (avoidance index >0). k-nearest neighbors (k-NN, with k=5) and decision trees were trained on 75% of the dataset (n=79 mice) and cross-validated (reaching 67-85% accuracy for k-NN and 90-95% for decision trees). Yet, classifiers performed at chance level on the test data set. We also tested other classification or regression indices (number of detours, latency-to-contact), yielding poorer results on the cross-validation dataset, and remaining at chance level on the test data set.

To conclude, this extensive analysis of mouse behavior at familiarization did not reveal the existence of key variables predicting subsequent learning and avoidance behavior on retrieval.

Supplementary Figures

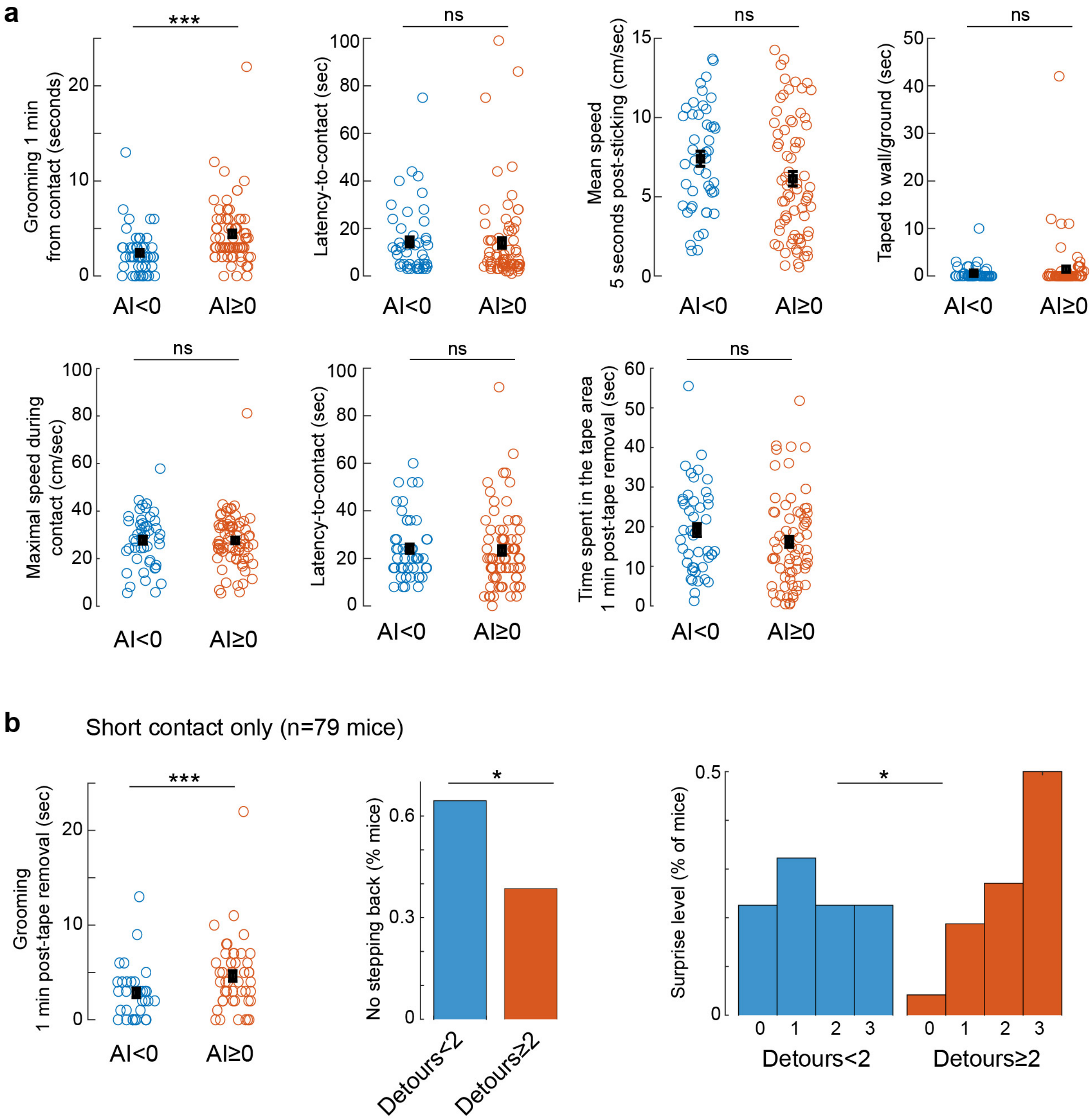

Supplementary Figure 1: Impact of behavioral features at familiarization on retrieval performance

Binary comparison of mice according to avoidance index or number of detours estimated at retrieval for all mice (n=118, **a**) or in the subset of mice with contact duration ≤30 seconds (n=79 mice, **b**). Time spent grooming 1 min from contact ($p<0.001$) or 1 min post-removal for short contact ($p<0.001$) were significantly different (independent t-tests). No stepping back at contact and low surprise levels were associated to low numbers of detours (Chi-square tests: $\chi2=4.68$, p=0.030 and $\chi2=10.8$, $p=0.013$, respectively).

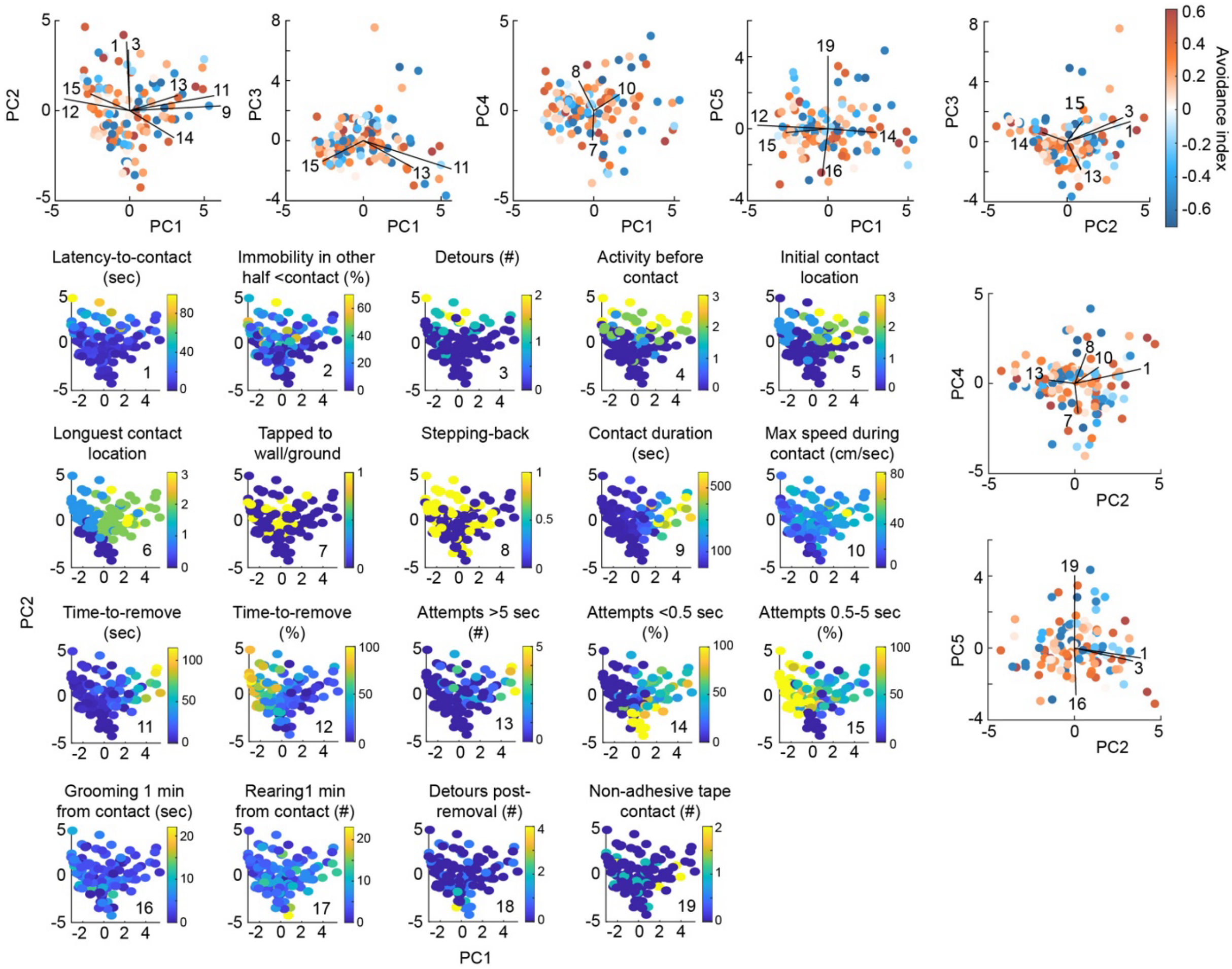

Supplementary Figure 2: Multidimensional analysis of familiarization and impact on retrieval Projections of the 19 parameters considered in familiarization along the first two principal components (parula colormap). Projections of the avoidance index (blue to red colormap) along with the contributions of key parameters represented by vectors on the first five principal components.

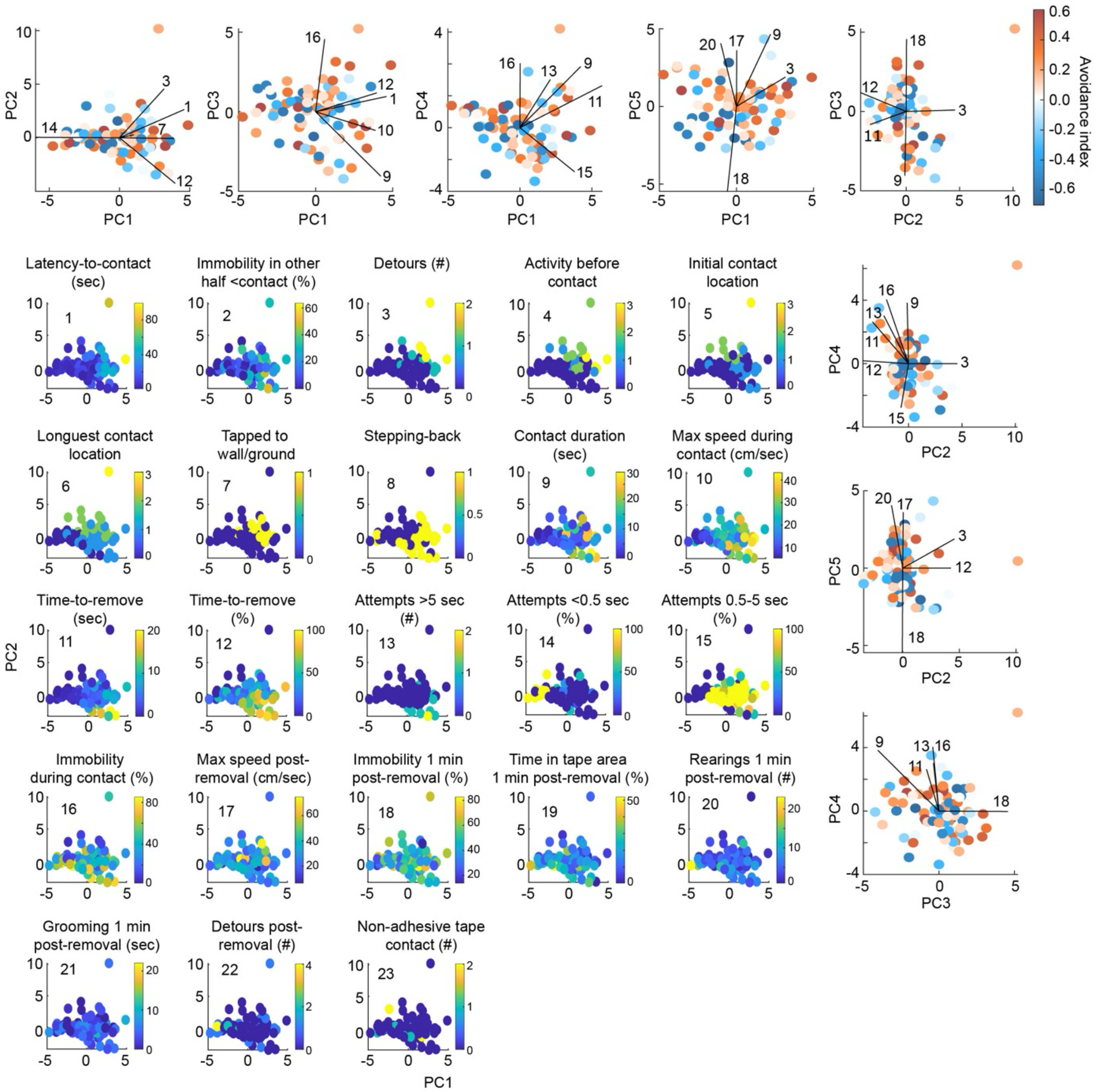

Supplementary Figure 3: Multidimensional analysis of familiarization and impact on retrieval for mice exhibiting short contact duration with the sticky tape

Projections of the 23 parameters considered in familiarization along the first two principal components (parula colormap) on mice exhibiting a short contact duration (≤30 seconds) with the sticky tape. Projections of the avoidance index (blue to red colormap) along with the contributions of key parameters represented by vectors on the first five principal components.
